# Prenatal Ethanol Exposure Results in Cell Type, Age, and Sex-Dependent Differences in the Neonatal Striatum That Coincide with Early Motor Deficits

**DOI:** 10.1523/ENEURO.0448-24.2025

**Published:** 2025-03-25

**Authors:** Adelaide R. Tousley, Ilana Deykin, Betul Koc, Pamela W. L. Yeh, Hermes H. Yeh

**Affiliations:** Department of Molecular and Systems Biology, Geisel School of Medicine at Dartmouth, Hanover, New Hampshire 03755

**Keywords:** alcohol, GABAergic interneurons, motor behavior, mouse model of FASD, spiny projection neurons

## Abstract

Delayed motor development is an early clinical sign of fetal alcohol spectrum disorders. However, changes at the neural circuit level that underlie early motor differences are underexplored. The striatum, the principal input nucleus of the basal ganglia, plays an important role in motor learning in adult animals, and the maturation of the striatal circuit has been associated with the development of early motor behaviors. Here, we briefly exposed pregnant C57BL/6 dams to ethanol (5% w/w) in a liquid diet on embryonic days 13.5–16.5 and assessed the mouse progeny using a series of nine brief motor behavior tasks on postnatal days 2–14. Live brain slices were then obtained from behaviorally tested mice for whole-cell voltage- and current-clamp electrophysiology to assess GABAergic/glutamatergic synaptic activity and passive/active properties in two populations of striatal neurons: GABAergic interneurons and spiny striatal projection neurons. Electrophysiologically recorded spiny striatal projection neurons were also filled intracellularly with biocytin for post hoc analysis of dendritic morphology. We found that prenatal ethanol exposure resulted in developmental motor delays that were more severe in male mice and coincided with sex-dependent differences in the maturation of striatal neurons. Our findings indicate that prenatal ethanol exposure results in dynamic morphological and functional changes to the developmental trajectories of striatal neurons commensurate with the development of motor behaviors that differ between male and female mice.

## Significance Statement

Developmental differences in motor behaviors are an early clinical sign of fetal alcohol spectrum disorders (FASDs) but the neural circuit-level changes that contribute to these differences have not yet been determined. Here we demonstrate that a brief binge exposure to ethanol alters the motor development of neonatal mice in a sex-dependent manner and identify concurrent differences in the functional, synaptic, and morphological development of striatal GABAergic interneurons and medium spiny striatal projection neurons. These data suggest that altered development of striatal neurons may contribute to differences in early motor development observed in individuals with FASD.

## Introduction

Developmental motor delays are among the earliest clinical symptoms observed in individuals diagnosed with fetal alcohol spectrum disorders (FASDs), an umbrella term encompassing the range of clinical diagnoses that may result from prenatal exposure to ethanol. Indeed, FASDs are the most common nongenetic cause of neurodevelopmental disorders worldwide (Lange et al., 2017; Wozniak et al., 2019). Individuals with FASD can develop motor differences including challenges with both gross and fine motor function, as well as sensorimotor integration (Doney et al., 2014, 2016; Lucas et al., 2016). However, changes at the level of neural circuits that contribute to early motor differences in individuals with FASD await elucidation.

As the principal input nucleus of the basal ganglia, the striatum contributes to motor learning in adult animals, and the maturation of the striatal circuit has been associated with the development of early motor behaviors (Dehorter et al., 2011; Graybiel and Grafton, 2015; Cataldi et al., 2021). Imaging studies indicate that prenatal ethanol exposure may modify both the size and functional connectivity of the developing striatum in individuals with FASD (Cortese et al., 2006; Mattson et al., 2011; Donald et al., 2016). Here, we investigated the effects of prenatal ethanol exposure on two populations of GABAergic striatal neurons, namely, GABAergic interneurons (GINs) and medium spiny striatal projection neurons (SPNs), asking how the altered maturation of these two populations of striatal neurons might relate to the development of motor behaviors during the first two postnatal weeks.

Despite comprising only a small proportion of striatal neurons (<1%), GINs play a critical role in regulating network activity in the striatum and in modulating striatal-mediated motor behaviors (Jin et al., 2014; Rueda-Orozco and Robbe, 2015; O’Hare et al., 2016; Xu et al., 2016; Lee et al., 2017; Martiros et al., 2018; Owen et al., 2018; Gazan et al., 2019; Gritton et al., 2019; Duhne et al., 2020; Holly et al., 2021). Prenatal ethanol exposure has been shown to alter the disposition and function of GINs in the adult striatum, and across the lifespan in several brain regions such as the cortex and hippocampus and in human postmortem tissue (De Giorgio et al., 2012; Skorput et al., 2015, 2019; Bengtsson Gonzales et al., 2020; Cuzon Carlson et al., 2020; Léger et al., 2020; Madden et al., 2020; Marguet et al., 2020). In contrast, direct and indirect pathway SPNs make up ∼95% of striatal neurons; increase and decrease motor behaviors, respectively; and, in concert, facilitate the onset of movement (Ferguson et al., 2011; Kravitz et al., 2012; Cui et al., 2013). Prenatal ethanol exposure can have a lasting impact on the morphology and function of SPNs in adult animals (Rice et al., 2012; Zhou et al., 2012; Cheng et al., 2018; Marquardt et al., 2020; Roselli et al., 2020). Although acute ethanol exposure has been shown to modify the function of striatal neurons in a subtype-specific manner in adult animals, how prenatal ethanol exposure may differentially affect striatal GINs and SPNs during early postnatal development has yet to be investigated (Blomeley et al., 2011; Marty and Spigelman, 2012; Patton et al., 2016).

We hypothesized that a brief binge-type exposure to ethanol in a liquid diet (5% w/w) from embryonic days (E) 13.5 to 16.5, a gestational period when GINs and SPNs are born in the ventral pallidum and actively migrate to populate the embryonic striatum, would alter the development of early motor behaviors in neonatal mice, as well as alter the functional, synaptic, and morphological development of striatal GINs and SPNs (Deacon et al., 1994; Olsson et al., 1998; Marin et al., 2000; Villar-Cerviño et al., 2015). We report here that prenatal ethanol exposure results in sex-dependent developmental motor differences concurrent with alterations in synaptic activity, passive/active electrical properties, and morphology of striatal neurons during the first two postnatal weeks.

## Materials and Methods

### Mice and prenatal ethanol exposure paradigm

**Figure 1.**
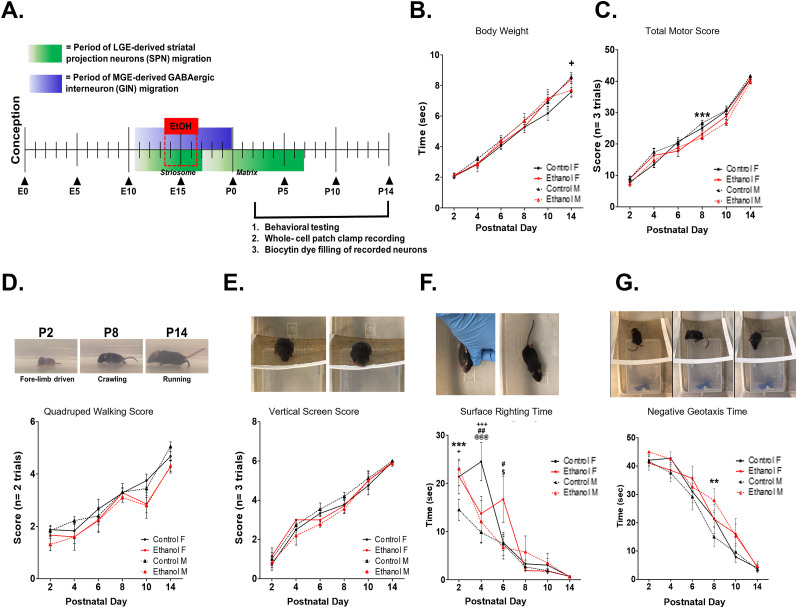
Experimental timeline and the influence of prenatal ethanol exposure on early postnatal sensorimotor development. ***A***, Pregnant dams were exposed to 5% (w/w) ethanol in a liquid diet, or a control (lab chow) diet from embryonic day (E) 13.5–16.5, a period of significant migration of early-born striosomal striatal projection neurons (SPNs) and striatal GABAergic interneurons (GINs), to the developing striatum. After birth at postnatal day (P) 0, mice were maintained to postnatal time points, P2, P4, P6, P8, P10, or P14, assessed for the development of a set of nine sensorimotor behaviors, and then killed for whole-cell patch-clamp recordings and morphological analysis of biocytin dye-filled cells. ***B***, A brief binge prenatal ethanol exposure does not alter the postnatal growth: body weight (g) of neonatal mice (3-way ANOVA: exposure: *F*_(1,197)_ = 0.387, *p* = 0.535, sex: *F*_(1,197)_ = 2.686, *p* = 0.103, postnatal day: *F*_(5,197)_ = 223.659, *p* < 0.001, exposure × sex × postnatal day: *F*_(5,197)_ = 1.416, *p* = 0.220, exposure × sex: *F*_(1,197)_ = 2.847, *p* = 0.093, exposure × postnatal day: *F*_(5,197)_ = 0.355, *p* = 0.879, sex × postnatal day: *F*_(5,197)_ = 0.222, *p* = 0.953). ***C***, Prenatal ethanol exposure resulted in decreased total motor score (TMS) in ethanol-exposed M mice, which significantly differed from control M mice at P8 (Kruskal–Wallis test, P2: *H*_(3)_ = 3.602, *p* = 0.308, P4: *H*_(3)_ = 4.883, *p* = 0.181, P6: *H*_(3)_ = 2.778, *p* = 0.427, P8: *H*_(3)_ = 11.343, *p* = 0.010, Dunn's post hoc test: ethanol M vs ethanol F: *p* = 0.009, P10: *H*_(3)_ = 0.000, *p* = 1.000, P14: *H*_(3)_ = 2.893, *p* = 0.408). ***D***, Prenatal ethanol exposure delayed the transition from forelimb-driven pivoting behavior, to crawling, and eventually running in ethanol-exposed M mice resulting in decreased quadruped walking scores (Kruskal–Wallis tests, P2: *H*_(3)_ = 4.509, *p* = 0.211, P4: *H*_(3)_ = 3.647, *p* = 0.301, P6: *H*_(3)_ = 1.407, *p* = 0.704, P8: *H*_(3)_ = 0.937, *p* = 0.817, P10: *H*_(3)_ = 3.568, *p* = 0.312, P14: *H*_(3)_ = 8.728, *p* = 0.033). ***E***, Prenatal ethanol exposure delayed the development of mature vertical screen task behavior in M mice (Kruskal–Wallis tests, P2: *H*_(3)_ = 0.731, *p =* 0.866, P4: *H*_(3)_ = 6.436, *p* = 0.096, P6: *H*_(3)_ = 7.248, *p* = 0.064, P8: *H*_(3)_ = 4.311, *p* = 0.230, P10: *H*_(3)_ = 0.602, *p* = 0.896, P14: *H*_(3)_ = 1.422, *p* = 0.700). ***F***, Prenatal ethanol exposure altered surface righting times in a sex-dependent manner (2-way ANOVA, group: *F*_(3,200)_ = 6.307, *p* = 0.0004, postnatal day: *F*_(5,200)_ = 81.71, *p* < 0.0001, group × postnatal day: *F*_(15,200)_ = 3.502, *p* < 0.001, Bonferroni’s post hoc tests: control M vs ethanol M: P2: *t* = 3.856, *p* < 0.001, ethanol M vs ethanol F: P6: *t* = 3.400, *p* < 0.01, control F vs control M: P2: *t* = 2.956, *p* < 0.05, P4: *t* = 5.498, *p* < 0.001, control M vs ethanol F: P4: *t* = 3.443, *p* < 0.01, P6: *t* = 3.226, *p* < 0.01, control F vs ethanol M: P4: *t* = 3.969, *p* < 0.001). ***G***, Prenatal ethanol exposure altered negative geotaxis time (2-way ANOVA, group: *F*_(3,200)_ = 3.502, *p =* 0.0164, postnatal day: *F*_(5,200)_ = 131.3, *p* < 0.001, group × postnatal day: *F*_(15,200)_ 0.7252, *p* = 0.7253, Bonferroni’s post hoc tests: P8: ethanol M vs control M, *t* = 3.447, *p* < 0.01). Data are presented as mean score or time, error bars are standard error of the mean (SEM), **p < 0.01, ***p < 0.001, control male versus ethanol male; ^##^*p* < 0.01, control female versus ethanol ^+^*p* < 0.05; ^+++^*p* < 0.001, control male versus control female; ^@@@^*p* < 0.001: control female versus ethanol. Data supported by Extended Data Figure 1-1.

All procedures involving mice were conducted in accordance with the National Institutes of Health *Guide for the Care and Use of Laboratory Animals* with the approval of the Institutional Animal Care and Use Committee (Protocol #00002109). Mice were housed on a 12 h light/dark cycle from 7 A.M. to 7 P.M. Nkx2.1Cre mice (The Jackson Laboratory, #008661) were crossed with Ai14Cre reporter mice (The Jackson Laboratory, #007914) on a C57BL/6 background, yielding offspring expressing a tdTomato reporter in embryonic GINs derived from the medial ganglionic eminence (MGE) beginning during embryonic development as diagramed in Figure 1*A*. Pregnant dams were fed 5% (w/w) ethanol in a liquid diet (L10251A, Research Diets) or a lab chow diet (5V5M, ScottPharma Solutions) from E13.5 to 16.5 with water available *ad libitum* (Xu et al., 2008). This ethanol exposure paradigm has been shown to routinely yield blood ethanol levels of ∼80 mg/dl (Skorput et al., 2015). Starting from birth, designated as postnatal day (P) 0, pups were cohoused with littermates and their female parent, maintained until designated behavioral testing days (P2, P4, P6, P8, P10, or P14), and then killed for electrophysiological experiments and morphological analyses.

10.1523/ENEURO.0448-24.2025.f1-1 Figure 1-1 **Prenatal ethanol exposure alters the development complex but not reflexive behaviors.** Prenatal ethanol exposure results in significant differences in **(A)** surface righting score **(B)** negative geotaxis score **(C)** horizontal screen score, **(D)** cliff avoidance score, that depend on both sex and postnatal age (Kruskall-Wallis tests, surface righting score: P2: H(3) = 6.102, p = 0.107, P4: H(3) = 8.660, p = 0.034, Dunn’s post-hoc tests: ethanol F vs. control F: p=0.042, P6: H(3) = 3.176, p = 0.365, P8: H(3) = 0.796, p = 0.850, P10: H(3) = 0.000, p = 1.000, P14: H(3) = 0.000, p = 1.000; negative geotaxis score: P2: H(3) = 1.319, p = 0.725, P4: H(3) = 1.566, p = 0.667, P6: H(3) = 1.633, p = 0.652, P8: H(3) = 12.926, p = 0.005, Dunn’s post-hoc tests: ethanol M vs. ethanol F: P8: p= 0.007, P10: H(3) = 3.064, p = 0.382, P14: H(3) = 6.392, p = 0.094; horizonal screen score: P2: H(3) = 0.000, p = 1.000, P4: H(3) = 0.000, p = 1.000, P6: H(3) = 5.348, p = 0.148, P8: H(3) = 2.145, p = 0.543, P10: H(3) = 1.534, p = 0.674, P14: H(3) = 1.012, p = 0.798; cliff avoidance score: P2: H(3) = 9.160, p 0.027, Dunn’s post-hoc, control F vs. control M: P2: p=0.017, P4: H(3) = 3.190, p = 0.363, P6: H(3) = 5.861, p = 0.119, P8: H(3) = 0.159, p = 0.984, P10: H(3) = 1.250, p = 0.741, P14: H(3) = 5.279, p = 0.152). No differences were observed between groups in **(E)** tactile startle score, **(F)** auditory startle score, **(G)** forepaw grasp score, **(H)** hindpaw grasp score. Data are presented as mean score or time, error bars are standard error of the mean (SEM), **p<0.01, control male vs. ethanol male; ^+^p<0.05, control male vs. control female; ^x^p<0.05, ethanol male vs. ethanol female. Download Figure 1-1, TIF file.

### Behavior

Between 9 A.M. and 1 P.M., on P2, P4, P6, P8, P10, or P14, mice of either sex were chosen at random and subjected to a series of tasks that assess sensorimotor behaviors, in a 14.5 cm × 25.5 cm × 10.5 cm clear plastic testing arena (Table 1; Fox, 1965). Mice completed three trials per task, excluding the quadruped walking task which was completed twice as animals noticeably decreased exploration of the testing arena between the second and third trials. Mice were allowed a 15–30 s break on a warming plate between each task and a 1 min break between each trial. Before each mouse was tested, the testing arena and materials were cleaned with Peroxigard (Virox Technologies, #29101). For behavioral tasks that assessed time to completion, surface righting time and negative geotaxis time, animals that failed to perform either behavior were assigned the maximum time for analysis: 30 and 45 s, respectively. To assess the overall performance of each mouse on all tasks, a total motor score (TMS) was computed as the sum of scores from the three trials (forepaw grasp, hindpaw grasp, tactile startle, auditory startle, horizontal screen, vertical screen, negative geotaxis, cliff avoidance) or two trials (quadruped walking) completed for each task. Performance on each task was then analyzed separately by postnatal age to determine which tasks contributed to the differences observed in TMS.

**Table 1.** Scoring parameters and description of neonatal motor behavioral testing tasks

Behavioral task	Trials	Scoring	Description
Surface righting	3	0 = absent, 1 = present; time to complete task (s)	Pup held on back for 5 s then given 30 s to right itself
Auditory startle	3	0 = absent, 1 = present	Pup presented with loud tone, observed for startle
Tactile startle	3	0 = absent, 1 = present	Pup presented with air puff, observed for startle
Grasp reflex	3	0 = absent, 1 = present	Pup stimulated on forepaw or hindpaw with dull side of metal blade, observed for grasp reflex
Horizontal screen test	3	0 = absent, 1 = grasp screen, 2 = lift screen	Pup pulled against horizontal wire mesh
Vertical screen test	3	0 = absent, 1 = grasp screen, 2 = climb screen	Pup pulled against vertical wire mesh
Negative geotaxis	3	0 = absent, 1 = orients to horizontal, 2 = orients to vertical, time to complete task (s)	Pup placed on 45° wire mesh head facing down, given 45 s to reverse direction and climb
Cliff avoidance	3	0 = absent, 1 = present	Mouse placed with snout and forepaw digits over a ledge (box), given up to 30 s to remiove
Quadruped walking	2	0 = no forward movement, 1 = crawling, asymmetric movements, 2 = crawling, symmetric movements, 3 = fast crawling/walking	Mouse recorded for 2 or 3 min in an empty cage

### Electrophysiology

Upon completion of behavioral experiments, all mice were asphyxiated with isoflurane, and acute coronal slices (250 µm) were prepared using a Leica VT1200s Vibratome (Leica Biosystems) beginning when the corpus callosum could be visualized connecting both hemispheres, posteriorly to when a fused anterior commissure could be discerned (4–6 slices per mouse depending on the developmental age). Slices were cut in oxygenated (95% O_2_, 5% CO_2_) cutting solution [in mM: 3 KCl, 7 MgCl_2_, 0.5 CaCl_2_, 1.25 NaH_2_PO_4_, 28 NaHCO_3_, 8.3 d-glucose, 110 sucrose, pH 7.4 (adjusted with 1 N NaOH)] and then maintained in artificial cerebrospinal fluid [aCSF; in mM: 124 NaCl, 5 KCl, 2 MgCl_2_, 2 CaCl_2_, 1.25 NaH_2_PO_4_, 26 NaHCO_3_, 10 d-glucose, pH 7.4 (adjusted with 1 N NaOH)], for a minimum of 1 h prior to electrophysiological recordings (Skorput et al., 2015; Delatour et al., 2019a,b; Tousley et al., 2022). For P2–P10 mice, slices were prepared in ice-cold cutting solution and then incubated for 20 min at 32°C followed by a 1 h incubation at room temperature (Delatour et al., 2019b). To optimize the survival of acute slices from P14 mice, slices were prepared in cutting solution at 32°C and maintained at 32°C in aCSF for at least 1 h prior to electrophysiological recording (Huang and Uusisaari, 2013).

Slices were placed in an acrylic recording chamber continuously perfused with oxygenated aCSF (0.5–1 ml/min) and maintained at 32°C. Striatal neurons were visualized with Hoffman modulation optics using a fixed-stage upright fluorescence microscope (Olympus BX41WI, Evident Corporation). Recording pipettes with resistances 8–10 MΩ were fabricated from borosilicate glass (Sutter Instrument; 1.5 mm; ID 0.86 mm) using a Flaming/Brown Micropipette Puller (Sutter Instrument, Model P80 PC). Whole-cell patch-clamp recordings were performed using a MultiClamp 700b amplifier (Molecular Devices), with signals low-pass filtered at 10 kHz (Clampex, version 9.2, Molecular Devices) and digitized at 25 kHz (Digidata 1320A, Molecular Devices).

Current-clamp recordings were conducted using a potassium gluconate-based internal solution (in mM): 100 K-gluconate, 2 MgCl_2_, 1 CaCl_2_, 11 EGTA, 10 HEPES, 30 KCl, 3 Mg + 2 ATP, 3 Na + GTP (adjusted to pH 7.3 with 1N KOH). Resting membrane potential (RMP) was determined immediately upon breaking into the cell membrane. Action potential (AP) firing rate was determined from a series of 8–10, 500 ms depolarizing current steps: P2 and P4–6 (0–80 pA by 10 pA), P8–10 (0–160 pA by 20 pA), and P14 (0–500 pA by 50 pA). Input resistance (IR) was calculated from a series of 8–10, 500 ms hyperpolarizing current steps: P2 and P4–6 (0 to −80 pA by 10 pA), P8–10 (0 to −160 pA by 20 pA), and P14 (0 to −500 pA by 50 pA). AP half-width and amplitude were determined from a single AP for each neuron: the second AP evoked by the threshold current was compared between neurons. AP threshold was determined as the current value when the slope (dV / dT) was >10 mV/ms. Capacitance and membrane time constant were calculated using a MultiClamp 700b commander with a 10 mV voltage step and a sampling rate of 0.4 kHz. Analysis of current-clamp recording data was conducted using Clampex 9.2 software (Molecular Devices).

Spontaneous postsynaptic currents (sPSCs) were monitored under whole-cell voltage clamp using a cesium methanesulfonate-based internal solution: 30 Cs-methanesulfonate, 10 HEPES, 0.5 EGTA, 8 NaCl, 10 Na-phosphocreatine, 4 Mg^2+^ ATP, and 0.4 Na^+^ GTP adjusted to pH 7.3 with 1 N CsOH, isolating glutamatergic synaptic currents by recording at a holding potential of −70 mV and GABAergic synaptic currents at a holding potential of 0 mV. Analysis of average frequency, amplitude, and charge of sPSCs from 2 min epochs of synaptic activity was performed using Mini Analysis software with manual confirmation of each event (version 6.0.7, Synaptosoft).

### Neuronal morphology

Internal solutions for both voltage-clamp and current-clamp recordings contained 2% Neurobiotin tracer (SP1120, VectorLabs), which filled neurons during whole-cell recordings (2–15 min). The filled cells were prepared for imaging and tracing as previously described (Delatour et al., 2019a,b; Tousley et al., 2022). Briefly, slices (250 μm) were fixed in 4% paraformaldehyde (PFA)/0.1 M phosphate-buffered saline (PBS) overnight and then maintained in 30% sucrose/0.1 M PBS prior to processing at 4°C. Slices were washed in 0.1 M PBS, then incubated for 30 min in 30% hydrogen peroxide (H_2_O_2_) in 0.1 M PBS followed by a 0.1 M PBS wash, and a second 30 min incubation in 30% H_2_O_2_ in 0.1 M PBS. Slices were then permeabilized and blocked in 10% NGS in 0.4% Triton X-100/0.1 M PBS for 2 h and then placed overnight at 4°C in 1:1,000 Dylight-488 streptavidin (#SA-5488-1, Vector Biosciences). Z-stack images of filled SPNs were captured at 20× magnification using a Zeiss LSM 510 laser-scanning confocal microscope (Zeiss, with a HENE 543 Laser using a Plan-Apochromat 20×/0.75 NA). Filled cells were traced and analyzed for soma area (µm^2^) and dendritic morphology: numbers of dendrites, mean nodes per dendrite, and mean dendritic length (µm). Sholl analysis was conducted using Neurolucida 360 software to assess the number of intersections per incremental 10 µm radius extending from the soma (version 2021.1.3, MBF Bioscience).

### Immunohistochemistry

Nkx2.1Cre × tdTomato mice were transcardially perfused with 4% PFA/0.1 M PBS. Brains were dissected and immersed overnight in 4% PFA/0.1 M PBS, followed by incubation for 1 d in 15% sucrose/0.1 M PBS, and then 1 d in 30% sucrose/0.1 M PBS. Cryosections (30 µm) were prepared using a sliding microtome and incubated overnight in 0.1 M PBS. Sections were permeabilized and blocked with 10% NGS in 0.25% Triton X-100/0.1 M PBS for 30 min and then incubated overnight at 4°C in 1:200 CTIP2 [25B6] primary antibody (ab18465, Abcam) in 0.1 M PBS. Sections were incubated overnight in 1:1,000 goat anti-rat Alexa Fluor-488 secondary antibody (A110006, Molecular Probes) at 4°C. Following an overnight incubation in 0.1 M PBS, sections were mounted and counterstained with 4′,6-diamidino-2-phenylindole (DAPI) and then coverslipped with FluorSave Reagent (#345789, Calbiochem). Images of fluorescent tdTomato+ striatal GINs and CTIP2+ SPNs were obtained using a CCD camera (Hamamatsu) mounted on an upright spinning disk confocal microscope with a 10× 0.30 NA objective (Olympus BX61WI, Evident). Digitized imaged were captured using Olympus cellSens software (version 1.18, Evident) and pseudocolored using FIJI (NIH; Schindelin et al., 2012).

### Statistical analysis

Three-way ANOVAs with experimental exposure (control vs ethanol), sex (female vs male), and postnatal day (for behavioral analyses, P2, P4, P6, P8, P10, and P14; for analyses of function and morphology, P2, P4–6, P8–10, and P14) as factors were performed using IBM SPSS (IBM SPSS Statistics for Windows, version 28.0). Where significant main effects or interactions were indicated, one-way ANOVAs comparing groups, control female, ethanol female, control male, and ethanol male, were performed for each postnatal age, followed by Bonferroni’s post hoc analyses or Kruskal–Wallis tests, with Dunn's post hoc analyses in cases where data were not normally distributed using GraphPad Prism software (GraphPad Prism, version 5.03). Normality was assessed using Shapiro–Wilk tests. For scored behavioral tasks and dendritic number, ordinal logistic regressions were performed using SPSS following assessment for multicollinearity, with Wald post hoc tests. Where significant exposure, sex, or postnatal main effects or interactions were determined, comparisons between groups were made at each designated postnatal day with Kruskal–Wallis tests and Dunn's post hoc analyses (IBM SPSS Statistics for Windows). Trends were reported where *p* values were <0.070.

Data were presented as mean ± standard error of mean (SEM). For all experiments, experimenters were blinded to experimental group. No more than one male and female animal per litter was used at a given time point, with animals from minimum of three litters used per time point. For electrophysiological recording experiments, cells were evenly sampled from all four striatal quadrants, dorsolateral, dorsomedial, ventrolateral, and ventromedial, with no more than one cell per quadrant per animal from at least three animals included for analysis. For behavioral experiments, 4–19 animals per group were assessed at each time point. Sample size for each experiment was determined based upon power analysis of preliminary data produced by our lab and previously published literature with the minimal number of animals used to obtain an *α* = 0.05 and 1-*β* = 0.8 (G*Power 3.1, Heinrich Heine University).

## Results

### A brief binge-type exposure to ethanol delays the development of motor behaviors in a sex-dependent manner

Both chronic and acute prenatal binge exposures to ethanol can result in developmental motor differences in rodent models (Fish et al., 1981; Molina et al., 1987; Schambra et al., 2015). We first asked if our model of a brief binge exposure to ethanol might alter the development of early motor responses to sensory stimuli. To evaluate early motor development, we employed a series of nine brief behavioral tasks first developed by Fox (1965) (Bignami, 1996; Crawley, 2012; Michetti et al., 2022; Table 1). Behavioral performance was assessed in male and female neonates on P2, P4, P6, P8, P10, or P14 (control: female: *N* = 63 mice, male: *N* = 70 mice; ethanol: female: *N* = 42, male: *N* = 46 mice; Table 2). Mice were assessed between P2 and P14, as P2 was when we first observed forelimb-driven pivoting behavior in some but not all neonates, while mice older than P14 were reluctant to complete several of the behavioral tasks, as previously reported (Armstrong et al., 2019). Animals were scored by blinded experimenters with higher scores indicating more mature behaviors (Table 1).

**Table 2.** Mice assessed for neonatal motor behaviors by postnatal age

Postnatal age	Control female	Control male	Ethanol female	Ethanol male
P2	8	12	5	13
P4	6	9	5	5
P6	9	10	4	9
P8	18	16	7	11
P10	10	9	7	5
P14	14	21	14	13

Our prenatal ethanol exposure did not result in gross differences in physical development insofar as the body weight (g) of behaviorally tested mice of either sex was unaltered over the first two postnatal weeks (Fig. 1*B*). Given these data, we asked if there was an overall effect of prenatal ethanol exposure on motor task performance and if that effect differed between female and male mice. A TMS was calculated for each tested animal as the sum of scores for each task over the course of three trials (forepaw grasp, hindpaw grasp, tactile startle, auditory startle, horizontal screen, vertical screen, negative geotaxis, cliff avoidance) or two trials (quadruped walking). Prenatal ethanol exposure resulted in significantly lower TMS indicative of delayed motor development (ordinal logistic regression, *p* < 0.001; Table 3, Fig. 1*C*). Additionally, while we did not observe an effect of biological sex on TMS, we identified a significant exposure × sex × postnatal day interaction (ordinal logistic regression, *p* < 0.001; Table 3). These findings suggest that prenatal ethanol exposure results in developmental motor differences that are dependent on both sex and the time point assessed.

**Table 3.** Neonatal motor behavioral task statistics

Effects	Total motor score	Quadruped walking score	Vertical screen score	Surface righting score	Surface righting time	Negative geotaxis score	Negative geotaxis time	Tactile startle score	Auditory startle score	Forepaw grasp score	Hindpaw grasp score	Horizontal screen score	Cliff avoidance score
Exposure (E)	Wald *χ*^2^_(1)_ = 13.975, ***p < 0.001	Wald *χ*^2^_(1)_ = 12.266, ***p < 0.001	Wald *χ*^2^_(1)_ = 0.000, *p* = 0.994	Wald *χ*^2^_(1)_ = 0.000, *p* = 1.000	*F*_(1,200)_ = 1.245, *p* = 0.266	Wald *χ*^2^_(1)_ = 0.000, *p* = 0.998	*F*_(1,200)_ = 6.660, *p = 0.011	Wald *χ*^2^_(1)_ = 0.000, *p* = 1.000	Wald *χ*^2^_(1)_ = 0.000, *p* = 1.000	Wald *χ*^2^_(1)_ = 0.000, *p* = 1.000	Wald *χ*^2^_(1)_ = 0.000, *p* = 1.000	Wald *χ*^2^_(1)_ = 0.000, *p* = 1.000	Wald *χ*^2^_(1)_ = 0.000, *p* = 0.999
Sex (S)	Wald *χ*^2^_(1)_ = 0.418, *p* = 0.518	Wald *χ*^2^_(1)_ = 0.140, *p* = 0.708	Wald *χ*^2^_(1)_ = 0.000, *p* = 1.000	Wald *χ*^2^_(1)_ = 0.000, *p* = 0.321	***F*_(1,200)_ = 9.424, *p* = 0.002**	Wald *χ*^2^_(1)_ = 0.000, *p* = 0.999	*F*_(1,200)_ = 0.016, *p* = 0.899	Wald *χ*^2^_(1)_ = 0.000, *p* = 1.000	Wald *χ*^2^_(1)_ = 0.000, *p* = 1.000	Wald *χ*^2^_(1)_ = 0.000, *p* = 1.000	Wald *χ*^2^_(1)_ = 0.000, *p* = 1.000	Wald *χ*^2^_(1)_ = 0.000, *p* = 1.000	Wald *χ*^2^_(1)_ = 0.000, *p* = 0.999
Postnatal day (P)	Wald *χ*^2^_(6)_ = 313.444, ***p < 0.001	*Wald χ* ^2^_(6)_ = 145.427, ***p < 0.001	*Wald χ* ^2^_(6)_ = 19.103, **p = 0.004	Wald *χ*^2^_(6)_ = 6.998, *p* = 1.000	*F*_(5/2000)_ = 81.703, **p < 0.001	*Wald χ* ^2^_(6)_ = 109.019, ***p < 0.001	***F*_(5,200)_ = 131.345, *p* < 0.0001**	Wald *χ*^2^_(6)_ = 0.000, *p* = 1.000	Wald *χ*^2^_(6)_ = 0.000, *p* = 1.000	Wald *χ*^2^_(6)_ = 0.000, *p* = 1.000	Wald *χ*^2^_(6)_ = 0.000, *p* = 1.000	**Wald *χ*^2^_(6)_ = 73.755, *p* < 0.001**	**Wald *χ*^2^_(6)_ = 79.290, *p* < 0.001**
E × S	Wald *χ*^2^_(1)_ = 7.355, **p = 0.007	Wald *χ*^2^_(1)_ = 0.713, *p* = 0.399	Wald *χ*^2^_(1)_ = 6.381, *p = 0.012	Wald *χ*^2^_(1)_ = 2.050, *p* = 0.152	*F*_(1,200)_ = 4,242, *p = 0.041	Wald *χ*^2^_(1)_ = 3.625, *p* = 0.057	*F*_(1,200)_ = 2.537, *p* = 0.113	Wald *χ*^2^_(1)_ = 0.000, *p* = 1.000	Wald *χ*^2^_(1)_ = 0.000, *p* = 0.997	Wald *χ*^2^_(1)_ = 0.000, *p* = 0.997	Wald *χ*^2^_(1)_ = 1.486, *p* = 0.223	Wald *χ*^2^_(1)_ = 0.909, *p* = 0.340	Wald *χ*^2^_(1)_ = 0.192, *p* = 0.661
E × P	Wald *χ*^2^_(5)_ = 2.204, *p* = 0.820	Wald *χ*^2^_(5)_ = 4.098, *p* = 0.535	Wald *χ*^2^_(5)_ = 2.994, *p* = 0.701	Wald *χ*^2^_(5)_ = 1.882, *p* = 0.865	*F*_(5,200)_ = 2.628 *p = 0.025	Wald *χ*^2^_(5)_ = 6.054, *p* = 0.301	*F*_(5,200)_ = 0.964, *p* = 0.441	Wald *χ*^2^_(5)_ = 0.000, *p* = 1.000	Wald *χ*^2^_(5)_ = 0.000, *p* = 1.000	Wald *χ*^2^_(5)_ = 0.000, *p* = 1.000	Wald *χ*^2^_(5)_ = 1.589, *p* = 0.903	Wald *χ*^2^_(5)_ = 1.662, *p* = 0.894	Wald *χ*^2^_(5)_ = 3.515, *p* = 0.621
P × S	Wald *χ*^2^_(5)_ = 5.999, *p* = 0.306	Wald *χ*^2^_(5)_ = 4.022, *p* = 0.546	Wald *χ*^2^_(5)_ = 2.238, *p* = 0.815	Wald *χ*^2^_(5)_ = 9.778, *p* = 0.082	*F*_(5,200)_ = 3.791, *p = 0.003	Wald *χ*^2^_(5)_ = 3.674, *p* = 0.597	*F*_(5,200)_ = 0.204, *p* = 0.961	Wald *χ*^2^_(5)_ = 0.000, *p* = 1.000	Wald *χ*^2^_(5)_ = 0.000, *p* = 1.000	Wald *χ*^2^_(5)_ = 0.000, *p* = 1.000	Wald *χ*^2^_(5)_ = 0.000, *p* = 1.000	Wald *χ*^2^_(5)_ = 1.501, *p* = 0.913	Wald *χ*^2^_(5)_ = 2.915, *p* = 0.713
E × S × P	Wald *χ*^2^_(24)_ = 323.717, ***p < 0.001	Wald *χ*^2^_(24)_ = 158.526, ***p < 0.001	Wald *χ*^2^_(24)_ = 97.034, ***p < 0.001	Wald *χ*^2^_(24)_ = 50.548, **p = 0.001	***F*_(5,200)_ = 4.128, *p* = 0.001**	Wald *χ*^2^_(24)_ = 154.322, ***p < 0.001	*F*_(5,200)_ = 1.046, *p* = 0.392	Wald *χ*^2^_(24)_ = 0.000, *p* = 1.000	Wald *χ*^2^_(24)_ = 0.958, *p* = 1.000	Wald *χ*^2^_(24)_ = 25.564, *p* = 0.376	Wald *χ*^2^_(24)_ = 0.072, *p* = 1.000	Wald *χ*^2^_(24)_ = 280.082, ***p < 0.001	Wald *χ*^2^_(24)_ = 97.404, ***p < 0.001

Statistical differences in performance on motor behavioral tasks completed by neonatal mice were determined with ordinal logistic regressions (scored behavioral tasks) or three-way ANOVAs (timed behavioral tasks). Major effects: exposure (control chow vs prenatal ethanol exposure embryonic day 13.5–16.5), sex (male vs female), and postnatal day (P2, P4, P6, P8, P10, and P14), with interactions: E × S (exposure × sex), E × P (exposure × postnatal day), P × S (postnatal day × sex), and E × S × P (exposure × sex postnatal day). Statistics with *p* value ≤0.05 are bolded.

Analysis of performance by task revealed that behavioral differences in mice exposed prenatally to ethanol were present in more complex tasks requiring coordinated movement and/or the integration of sensory and motor information: with an overall effect on quadruped walking score (Fig. 1*D*) and exposure × sex × postnatal day interactions for quadruped walking score, vertical screen score (Fig. 1*E*), surface righting score (Extended Data Fig. 1-1*H*), negative geotaxis score (Extended Data Fig. 1-1*G*), horizontal screen score (Extended Data Fig. 1-1*C*), and cliff avoidance score (Extended Data Fig. 1-1*F*; ordinal logistic regression, exposure: quadruped walking: *p* < 0.001, vertical screen: *p* < 0.001; exposure × sex × postnatal day: quadruped walking: *p* < 0.001, vertical screen: *p* < 0.001, surface righting score: *p* = 0.001, negative geotaxis: *p* = 0.001, horizontal screen: *p* ≤ 0.001, cliff avoidance score: *p* < 0.001; Table 3). Those behavioral tasks driven by simple reflex loops, such as tactile startle score (Extended Data Fig. 1-1*A*), auditory startle score (Extended Data Fig. 1-1*D*), forepaw grasp score (Extended Data Fig. 1-1*B*), and hindpaw grasp score (Extended Data Fig. 1-1*E*), were unchanged in mice with prenatal ethanol exposure (Table 3).

We next asked when behavioral differences might be most pronounced between groups, control female, ethanol female, control male, and ethanol male, during the first two postnatal weeks. We found that prenatal ethanol exposure resulted in significantly lower TMS in male mice at P8 relative to control-fed male mice (Fig. 1*C*). While no significant between group differences were identified at a single postnatal day in scoring of quadruped walking behavior, the most distinct between group differences were observed at P14, when animals are making the progression from immature crawling behavior involving all four limbs (score = 2), to running, indicated by an elevated trunk, decreased hindlimb slips, more synchronous fore and hindlimb movements, and a faster overall speed (score, 3), with fewer ethanol-exposed male mice demonstrating running behavior (31 vs 68% in control-fed males; Fig. 1*D*; Fox, 1965; Altman and Sudarshan, 1975). Differences were less pronounced during the onset of early postnatal pivoting behavior, involving only the use of forelimbs (score, 1), or in the transition from pivoting to crawling (Fig. 1*D*). The trends toward between group differences in vertical screen task performance were also the most pronounced at P6, when animals are first able to grasp and hold their position on the vertical screen (score, 1) but before they are able to climb the vertical screen (score, 2), with ethanol-exposed male mice again demonstrating the least mature behaviors (*p* = 0.064; Fig. 1*E*).

In contrast to scored behavioral tasks which allowed us to assess the absence or presence of early motor behaviors across development, we also assessed the time it took mice to complete negative geotaxis and surface righting behaviors to determine if more subtle alterations in motor behavior might be present but not accounted for by observer scoring (Extended Data Fig. 1-1*G,H*). Unlike the sex-dependent effects of prenatal ethanol exposure on negative geotaxis score, we determined that prenatal ethanol exposure significantly increased negative geotaxis times in both female and male mice, and analysis of between group differences at individual postnatal days suggested that the prenatal ethanol exposure resulted in significantly increased negative geotaxis times in male mice relative to control-fed male mice at P8 (3-way ANOVA, exposure: *p* = 0.011; Fig. 1*G*, Table 3).

Similarly, although comparison of surface righting scores did not reveal a significant main effect of biological sex, comparison of surface righting times did demonstrate significant effects of age and sex-dependent effects of ethanol exposure with age-matched female mice generally demonstrating more mature behaviors than male mice in both ethanol and control-fed mice that depended on the postnatal day assessed (three-way ANOVA: sex: *p* = 0.002; exposure × sex: *p* = 0.041, sex × postnatal day: *p* = 0.003; Fig. 1*F*, Extended Data Fig. 1-1*H*, Table 3). Comparisons of surface righting times between groups also suggested that prenatal ethanol exposure may differentially alter developmental trajectories of surface righting behavior in male and female mice (three-way ANOVA: exposure × postnatal day: *p* = 0.025; exposure × sex × postnatal day: *p* = 0.001). Prenatal ethanol exposure resulted in increased surface righting times in male mice relative to control-fed male mice at P2. However, the effects of prenatal ethanol exposure on surface righting time in female mice were more complex (Fig. 1*F*). Although ethanol-exposed female mice demonstrated increased surface righting times relative to control-fed and ethanol-exposed male mice at P6, this was not consistent across early postnatal development. At P4, ethanol-exposed female mice, as well as ethanol-exposed and control-fed male mice, demonstrated decreased surface righting times when compared with those of control-fed female mice (Fig. 1*F*). These data suggest that prenatal ethanol exposure can result in both improved performance (P4) and in deficits (P6) in surface righting behavior in female mice depending on the postnatal day.

In summary, prenatal ethanol exposure resulted in delayed development of motor behaviors in male mice, while the effects of exposure on female mice differed depending upon both the age of the mice and the behavior assessed. The delays in motor development observed in male animals were apparent at multiple postnatal ages (P2, P8, P14) and found particularly in tasks requiring coordinated motor responses (quadruped walking, vertical screen, surface righting, and negative geotaxis times) rather than simple tasks requiring reflexive behaviors. Differences in behaviors are subtle and made more evident when assessed using quantitative (surface righting time) rather than qualitative methods of assessment (surface righting score).

### Prenatal ethanol exposure differentially alters the maturation of active and passive properties of striatal neurons in male and female mice

**Figure 2.**
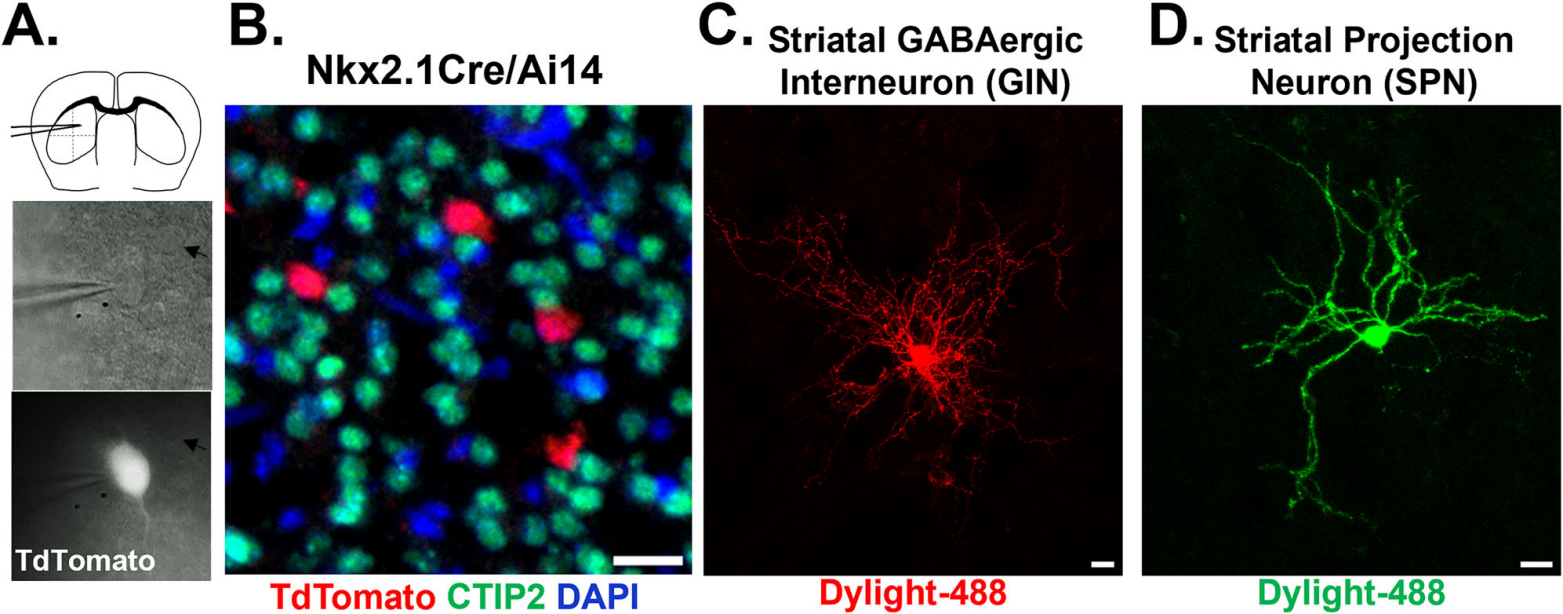
Identifying striatal GABAergic interneurons (GINs) and striatal projection neurons (SPNs) in acute cortical slices from Nkx2.1Cre × TdTomato mice. ***A***, Striatal GINs and SPNs were identified during whole-cell patch-clamp recordings from 250 µm acute coronal slices from Nkx2.1Cre × Tdtomato mice based on tdTomato (red) expression in MGE-derived GABAergic interneurons. Top, Schematic of whole-cell patch-clamp recordings from striatal neurons; middle, 40× magnification Hoffman modulated contrast image of acute slice during recording from a striatal GIN and a neighboring SPN (arrow); bottom, fluorescent tdTomato + striatal GIN (white) and a neighboring SPN (arrow). ***B***, A SPN-specific nuclear immunomarker CTIP2 (green) does not colabel td-Tomato + GINs (red) in the dorsal striatum of a P6 Nkx2.1Cre mice, with a DAPI-counterstain (blue). ***C***, Image of a neurobiotin-filled P14 striatal GIN after recording, pseudocolored (red). ***D***, Image of a neurobiotin-filled P14 SPN after recording (green). Scale bars, 20 µm.

**Figure 3.**
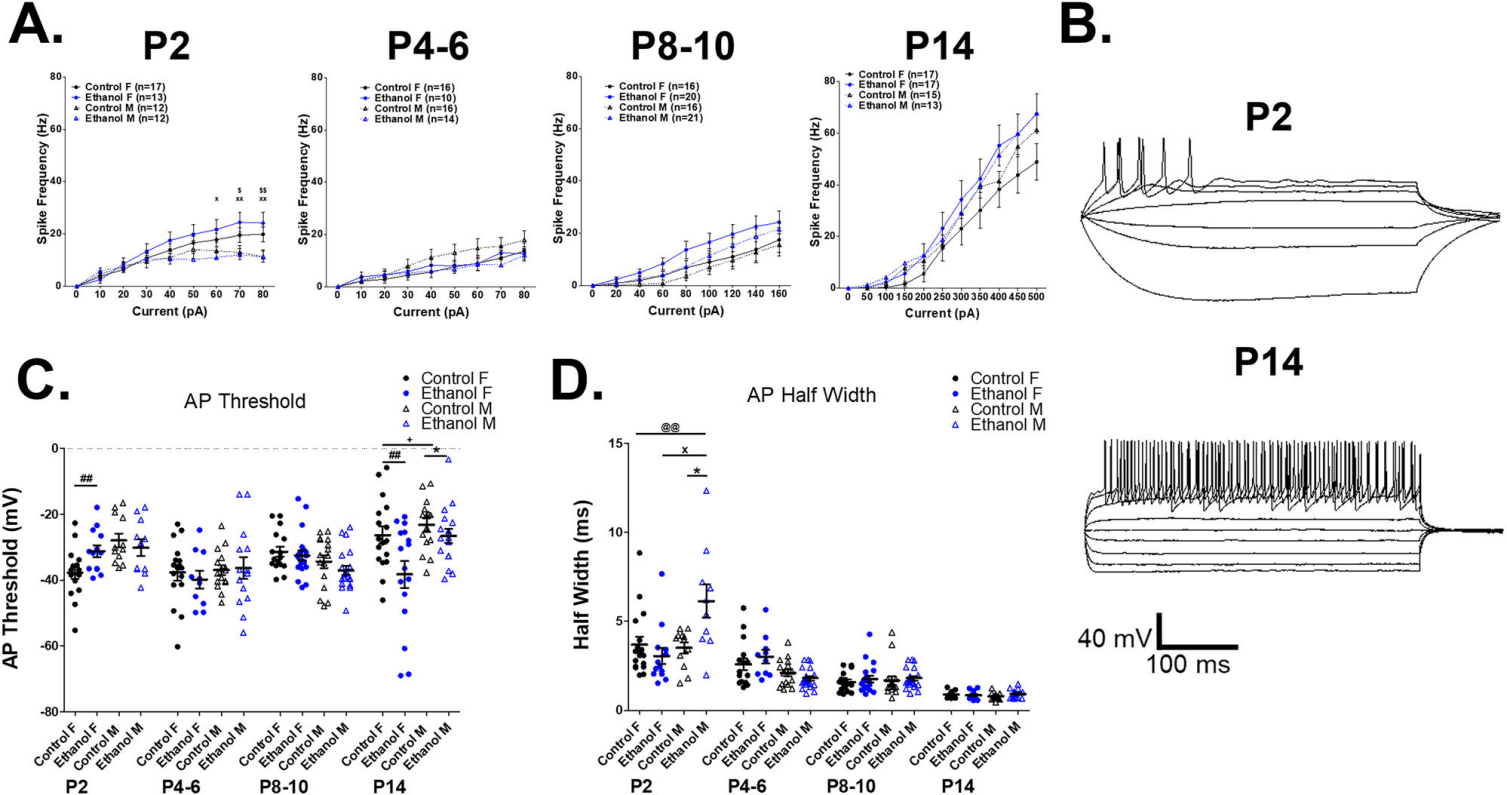
Prenatal ethanol exposure alters the functional development of striatal GABAergic interneurons (GINs) in early postnatal mice in an age- and sex-dependent manner. ***A***, Prenatal ethanol exposure modifies the firing rate (Hz) of striatal GIN in an age- and sex-dependent manner. At P2, striatal GINs from ethanol-exposed F mice had higher firing rates relative to those from ethanol-exposed and control-fed M mice (2-way ANOVA, group: *F*_(3,450)_ = 10.56, *p* < 0.0001, current: *F*_(8,450)_ = 24.52, *p* < 0.0001, group × current *F*_(24,450)_ = 1.282, *p* = 0.1691, Bonferroni’s post hoc tests: ethanol F vs ethanol M: 60 pA: *t* = 2.981, *p* < 0.05, 70 pA: *t* = 3.474, *p* < 0.01, 80 pA: *t* = 3.620, *p* < 0.01; ethanol F vs control M: 70 pA: *t* = 3.242, *p* < 0.05, 80 pA: *t* = 3.574, *p* < 0.01). At P4–6, firing rate significantly differed between groups with striatal GINs: control-fed M mice demonstrated higher firing rates relative to those from ethanol-exposed M and F and control-fed M mice (2-way ANOVA, group: *F*_(3,468)_ = 4.934, *p* = 0.0022, current: *F*_(8,468)_ = 14.26, *p* < 0.001, group × current: *F*_(24,468)_ = 0.4322, *p* = 0.9922). At P8–10 and P14, firing rate again significantly differed between groups with striatal GINs from ethanol-fed mice demonstrating higher firing rates relative to those from control-fed mice (2-way ANOVAs, P8–10: group: *F*_(3,621)_ = 11.15, *p* < 0.0001, current: *F*_(8,621)_ = 28.31, *p* < 0.0001, group × current: *F*_(24,621)_ = 0.4459, *p* = 0.9904; P14: group: *F*_(3,726)_ = 4.871, *p* = 0.0023, current: *F*_(10,726)_ = 56.96, *p* < 0.0001, group × current: *F*_(30,726)_ = 0.3229, *p* = 0.9998). ***B***, Example traces of voltage responses of striatal GINs following depolarizing current steps from control-fed female mice at P2 and P14. ***C***, Prenatal ethanol exposure significantly depolarized AP threshold in GINs from F mice relative to those from control-fed F mice at P2, hyperpolarized AP threshold in GINs from F and M mice relative to control-fed F and M mice at P14, and control-fed F relative to control-fed M mice but did not alter AP threshold from P4–10 (1-way ANOVAs, P2: *F*_(3,50)_ = 4.298, *p* = 0.005, Bonferroni’s post hoc tests: ethanol F vs control F, *p* = 0.005, P4–6: *F*_(3,50)_ = 0.949, *p* = 0.424, P8–10: *F*_(3,69)_ = 2.859, *p* = 0.043, P14: *F*_(3,69)_ = 5.352, *p* = 0.002 Bonferroni’s post hoc tests: ethanol F vs control F, *p* = 0.003, ethanol M vs control M: *p* = 0.027, control F vs control M: *p* = 0.023). ***D***, Prenatal ethanol exposure significantly increased GIN AP half-width in M mice relative to those from control-fed M or F, or ethanol-exposed F mice at P2, but did not affect GIN AP half-width P4–14 (1-way ANOVAs, P2: group: *F*_(3,50)_ = 5.639, *p* = 0.002, Bonferroni’s post hoc tests: ethanol M vs control M, *p* = 0.002, ethanol M vs ethanol F, *p* = 0.014, ethanol M vs control F, *p* = 0.014; P4–6 *F*_(3,50)_ = 1.335, *p* = 0.273; P8–10 *F*_(3,69)_ = 0.699, *p* = 0.556; P14: *F*_(3,61)_ = 1.086, *p* = 0.362). Data are presented as means (bars), error bars are standard error of the mean (SEM), dots are individual neurons from at least three animals per group. *p < 0.05, control male versus ethanol male; ^##^*p* < 0.01, control female versus ethanol female; ^+^*p* < 0.05, control male versus control female; ^@@^*p* < 0.01, control female versus ethanol male; ^$^*p* < 0.05, ^$$^*p* < 0.01, control male versus ethanol female; ^x^*p* < 0.05, ^xx^*p* < 0.01, ethanol male versus ethanol female. Data supported by Extended Data Figures 3-1, 3-2.

**Figure 4.**
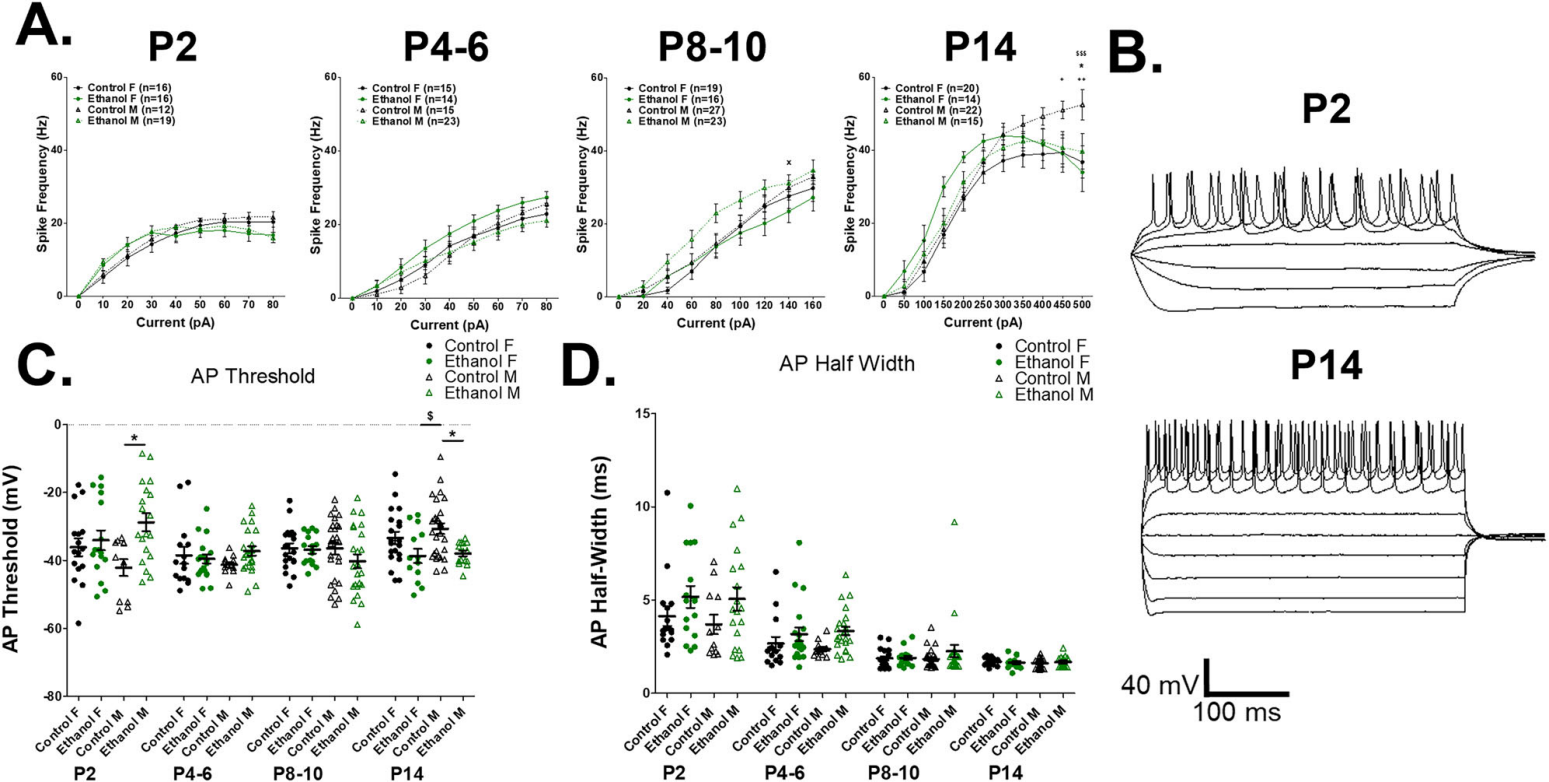
Prenatal ethanol exposure alters the functional development of striatal spiny projection neurons (SPNs) in early postnatal mice in an age- and sex-dependent manner. ***A***, Prenatal ethanol exposure modifies the firing rate of SPN in an age- and sex-dependent manner. While no significant differences were observed between groups: control F, ethanol F, control M, and ethanol M at P2, group-dependent differences were observed at P4–6, P8–10, and P14, with group differences varying based on current input at P14 (2-way ANOVAs, P2: *F*_(3,531)_ = 0.6138, *p* = 0.6063, current: *F*_(8,531)_ = 44.82 *p* < 0.0001, group × current: *F*_(8,531)_ = 0.7447, *p* = 0.8059; P4–6: group: 2-way ANOVA, *F*_(3,567)_ = 6.923, *p* = 0.0001, current: *F*_(8,567)_ = 81.60, *p* < 0.001, group × current: *F*_(24,567)_ = 0.7128, *p* = 0.8409; P8–10: group: *F*_(3,729)_ = 12.97, *p* < 0.001, current: *F*_(8,729)_ = 99.70, *p* < 0.001, group × current: *F*_(24,729)_ = 0.6896, *p* = 0.8647; P14: group: *F*_(3,803)_ = 7.874, *p* < 0.001, current: *F*_(8,803)_ = 104.4, *p* < 0.0001, group × current: *F*_(24,803)_ = 1.676, *p* = 0.0136). At P8, prenatal ethanol exposure significantly increased SPN firing rate in M mice relative to ethanol-exposed F mice. At P14, prenatal ethanol exposure significantly decreased SPN firing rate in M mice relative to control-fed M mice, while control-fed M mice demonstrated higher firing rates than both control-fed and ethanol-exposed F mice (Bonferroni’s post hoc tests, ethanol M vs control M: 500 pA: *t* = 2.864, *p* < 0.05, control M vs control F: 450 pA: *t* = 2.854, *p* < 0.05, 500 pA: *t* = 3.856, *p* < 0.01, control M vs ethanol F: 500 pA: *t* = 4.050, *p* < 0.001). ***B***, Example traces of voltage responses of striatal GINs following hyperpolarizing and depolarizing current steps from control-fed female mice at P2 and P14. ***C***, Prenatal ethanol exposure significantly depolarized AP threshold at P2 and hyperpolarized at P14 in SPNs from M mice, relative to those from control-fed M mice, and in depolarized AP threshold in SPNs from F mice relative to control-fed M mice at P14 but did not alter AP threshold between P4 and 10 (1-way ANOVAs, P2: *F*_(3,59)_ = 3.862, *p* = 0.014, Bonferroni’s post hoc test: ethanol M vs control M: *p* 0.009; P4–6: *F*_(3,66)_ = 1.039, *p* = 0.381; P8–10: *F*_(3,81)_ = 1.180, *p* = 0.323; P14: *F*_(3,73)_ = 5.055, *p* = 0.003, Bonferroni’s post hoc tests: ethanol M vs control M: *p* 0.020, ethanol F vs control M: *p* = 0.010). ***D***, Prenatal ethanol exposure did not alter the SPN AP half-width (1-way ANOVAs, P2: *F*_(3,59)_ = 1.384, *p* = 0.257, P4–6: *F*_(3,66)_ = 1.979, *p* = 0.126, P8–10: *F*_(3,81)_ = 1.017, *p* = 0.389, P14: *F*_(3,73)_ = 0.419, *p* = 0.740). Data are presented as means (bars), error bars are standard error of the mean (SEM), dots are individual neurons from at least three animals per group. *p < 0.05, control male versus ethanol male; ^+^*p* < 0.05, ^++^*p* < 0.01, control male versus control female; ^$^*p* < 0.05,^$$$^*p* < 0.001, control male versus ethanol female; ^x^*p* < 0.05, ethanol male versus ethanol female. Data supported by Extended Data Figures 4-1, 4-2.

Concurrent with the onset of increasingly complex motor behaviors during the first postnatal month, striatal GINs and SPNs gradually develop adult-like firing and membrane properties (Tepper et al., 1998; Belleau and Warren, 2000; Plotkin et al., 2005; Chesselet et al., 2007). These include changes in the characteristics of APs: increased AP firing rate, and decreased half-width, as well as shifts in membrane properties that confer decreased neuronal excitability, notably more depolarized RMP and AP threshold, and decreased IR. The development of mature quadruped walking behavior has been closely associated with the functional maturation of SPNs in neonates during this postnatal time period (Dehorter et al., 2011). How striatal GINs mature during this period, and how the development of striatal GINs and SPNs may be altered in early postnatal development following a prenatal ethanol exposure, has yet to be explored. Thus, we asked if prenatal ethanol exposure might alter the functional development of striatal GINs and SPNs. To differentiate striatal GINs and SPNs in acute slices during whole-cell patch-clamp recording experiments, we crossed the Nkx2.1Cre mouse line with a Ai14Cre reporter mice, resulting in tdTomato expression in MGE-derived striatal GINs (Xu et al., 2008; Fig. 2*A*,*B*). In addition, we confirmed the identity of SPNs and GINs based on their distinctive morphological properties by filling cells with 2% neurobiotin dye during whole-cell recording and by their distinctive firing properties (Figs. 2*A–D*, 3*B*, 4*B*).

#### Striatal GINs: AP firing properties

We first asked if prenatal ethanol exposure altered intrinsic excitability of striatal GINs by assessing the firing rate and AP characteristics (AP threshold, AP half-width, or AP peak amplitude) in response to a series of depolarizing current steps: P2 and P4–6 (0–80 pA by 10 pA), P8–10 (0–160 pA by 20 pA), and P14 (0–500 pA by 50 pA). At P2, striatal GINs from female mice displayed an increased firing rate regardless of group (Fig. 3*A*). However, only GINs from ethanol-exposed female mice had firing rates that significantly differed from ethanol-exposed or control-fed male mice. The firing rate of striatal GINs from ethanol-fed female mice did not differ from control-fed female mice (Fig. 3*A*). At P4–6, firing rate differed significantly between groups, with the highest firing rates observed in striatal GINs from control-fed male mice which were increased relative ethanol-exposed males and females, as well as control females (Fig. 3*A*). At P8–10, GIN firing rate again differed significantly between groups, with the largest differences in firing rate observed between striatal GINs in the ethanol-exposed and control-fed female and male cohorts (Fig. 3*A*).

10.1523/ENEURO.0448-24.2025.f3-1 Figure 3-1 **Prenatal ethanol exposure differentially effects the intrinsic properties of striatal GINs from female and male mice, depending on the postnatal day.** **(A)** IV curves for responses to hyperpolarizing current steps during whole-cell current clamp recordings of striatal GINs during the first postnatal week (2-way ANOVAs, P2: group: F(3,450)= 7.015, p=0.=0001, current: F(8,450) = 63.35, p<0.001, group x current: F(24,450)=0.2834, p=0.9996; P4-6: group: F(3,468)= 11.35, p<0.001, current: F(8,468) = 38.60, p<0.001, group x current: F(24,468)=0.2114, p=1.000; P8-10: group: F(3,621)= 25.22, p<0.001, current: F(8,621) = 74.29, p<0.001, group x current: F(24,621)=0.2189, p=0.9890; P14: group: F(3,671)= 15.53, p<0.001, current: F(10,671)= 83.66, p<0.001, group x current: F(30,671)=0.08655, p=1.000. **(B)** Prenatal ethanol exposure resulted in sex-dependent differences in resting membrane potential (RMP) that varied based on the postnatal day, though no significant differences were observed between groups on individual postnatal days (1 way ANOVAs, P2: F(3,50)= 2.008, p=0.125, P4-6: F(3,50)= 1.293, p=0.287, P8-10: F(3,69)= 2.244, p=0.091, P14: F(3,61)=0.642, p=0.591). **(C)** Input resistance (IR) (mΩ) and **(D)** membrane time constant (ms) were unaffected in striatal GINs were unaffected by prenatal ethanol exposure, sex or postnatal day (1-way ANOVAs, IR: P2: F(3,50) = 0.611, p =0.649 P4-6: F(3,50) = 0.219, p =0.883; P8-10: F(3,69) = 0.557, p =0.302; P14: F(3,61) = 0.611, p =0.610; membrane time constant: P2: F(3,50) = 1.318, p=0.279; P4-6: F(3,61) = 0.689, p =0.563; P8-10: F(3,69) = 1.239, p =0.302; P14: F(3,61) = 1.277, p =0.294). Data are presented as means (bars), error bars are standard error of the mean (SEM), dots are individual neurons from at least 3 animals per group.^ $^p<0.05, control male vs. ethanol female. Download Figure 3-1, TIF file.

10.1523/ENEURO.0448-24.2025.f3-2 Figure 3-2 **Prenatal ethanol exposure does not alter the action potential (AP) amplitude of developing striatal GABAergic interneurons (GINs) or striatal projection neurons (SPNs).** **(A)** The peak amplitude of APs (pA) did not differ in striatal GINs or **(B)** SPNs following prenatal ethanol exposure. Data are presented as means (bars), error bars are standard error of the mean (SEM), dots are individual neurons from at least 3 animals per group. Download Figure 3-2, TIF file.

Prenatal ethanol exposure also differentially altered the AP threshold and AP half-width of developing striatal GINs from male and female mice depending on the postnatal day (three-way ANOVAs, AP threshold: exposure × sex × postnatal day: *p* = 0.021, AP half-width: exposure × sex × postnatal day: *p* < 0.001; Table 2; Fig. 3*C*,*D*). At P2, prenatal ethanol exposure significantly altered both AP half-width and AP threshold (Fig. 3*C*,*D*). Prenatal ethanol exposure significantly increased AP half-width in striatal GINs recorded from male mice, relative to control-fed male mice (Fig. 3*D*). AP threshold was more depolarized in striatal GINs recorded from ethanol-exposed female mice relative to control-fed female mice, while there was only a trend toward a similar depolarization of AP threshold in those from ethanol-exposed male mice, relative to those from control-fed male mice (*p* = 0.061; Fig. 3*C*). By the end of the second postnatal week (P14), prenatal ethanol exposure resulted in hyperpolarized AP thresholds in striatal GINs recorded from both female and male mice relative to control-fed female and male mice, respectively (Fig. 3*C*). The peak amplitude of APs recorded from striatal GINs was unaffected by prenatal ethanol exposure (Extended Data Fig. 3-1*A*).

These data suggest that prenatal ethanol exposure differentially alters the development of striatal GINs in male and female mice. During early postnatal development, striatal GINs in female mouse pups exposed prenatally to ethanol developed early increases in AP firing, despite more depolarized AP thresholds that transiently resolved at P4–6, but were again present at P8–10 and P14 (Fig. 3*A*,*C*). In contrast, striatal GINs from male mice failed to mature appropriately following prenatal ethanol exposure, as indicated by wider APs at P2, and an absence of increased GIN firing rate at P4 (Fig. 3*A*,*D*). However, by the end of the first postnatal week striatal GINs from both male and female mice displayed an increased excitability with increased firing rates and more hyperpolarized AP thresholds (Fig. 3*A*,*C*).

#### SPNs: AP firing properties

As we found in striatal GINs, prenatal ethanol exposure resulted in differential effects on SPN AP firing rate depending on sex and postnatal age (Fig. 4*A*). At P2, prenatal ethanol exposure had no significant effect on AP firing rate when SPNs from control-fed and ethanol-exposed cohorts of either sex were compared (Fig. 4*A*). However, significant between group differences were observed at P4–6, P8–10, and P14 (Fig. 4*A*). At P4–6, prenatal ethanol exposure resulted in an increased firing rate in SPNs from female mice, relative to those from ethanol-exposed male mice (Fig. 4*A*). In contrast, at P8–10, SPNs from ethanol-exposed male mice displayed an increase in firing rate relative to those from ethanol-exposed female mice (Fig. 4*A*). At P14 the effects of prenatal ethanol exposure differed between groups depending on the size of the current step. At low levels of current input, SPNs from ethanol-exposed female mice displayed a higher firing rate (Fig. 4*A*). With increasing depolarizing current steps, SPNs from male mice exposed prenatally to ethanol displayed lower firing rates relative to control-fed male mice (Fig. 4*A*). SPNs from both control-fed and ethanol-exposed female mice also had lower firing rates compared with those from control-fed male mice.

10.1523/ENEURO.0448-24.2025.f4-1 Figure 4-1 **Prenatal ethanol exposure differentially effects the intrinsic properties of striatal SPNs from female and male mice, depending on the postnatal day.** **(A)** The effects of prenatal ethanol exposure on IV curves for responses to hyperpolarizing current steps during whole-cell current clamp recordings of SPNs during the first postnatal week vary by group (2-way ANOVAs, P2: group: F(3,531)= 3.047, p=0.0284, current: F(8,531) = 151.8, p<0.001, group x current: F(24, 531)=0.2490, p=0.9999; P4-6: group: F(3,540)= 13.10, p<0.001, current: F(8,540) = 169.0, p<0.001, group x current: F(24, 540)=0.4369, p=0.9999; P8-10: group: F(3,765)= 14.67, p<0.001, current: F(8,765) = 164.9, p<0.001, group x current: F(24,765)=0.4551, p=0.9890; P14: group: F(3,770)= 3.537, p=0.0145, current: F(10,770) = 243, p<0.001, group x current: F(30,770)=0.1753, p=1.000). **(B)** Prenatal ethanol exposure resulted in sex-dependent differences in SPN RMP that varied based on the postnatal day: At P4-6: prenatal ethanol exposure resulted in significantly depolarized RMP in male mice relative to control-fed male and female mice (1-way ANOVA: F(3,66) = 4.632, p=0.005, Bonferroni post-hoc tests: ethanol M vs. control F: p=0.023**,** ethanol M vs control F: p=0.004). SPN RMP was unaltered by prenatal ethanol exposure at P2, 8-10 or P14 (1-way ANOVAs, P2: F(3,59) = 1.790, p =0.159; P8-10: F(3,81) = 0.796, p =0.499; P14: F(3,73) = 1.042, p = 0.379). **(C)** Prenatal ethanol exposure did not alter the IR of SPNs (1-way ANOVAs, P2: F(3,59) = 0.946, p =0.424; P4-6: F(3,66) = 1.039, p =0.202; P8-10: F(3,81) = 1.182, p =0.322; P14: F(3,73) = 1.034, p=0.383). **(D)** Prenatal ethanol exposure results in decreased membrane time constant in SPNs from ethanol-exposed M mice at P8-10, relative to control-fed F mice, but did not alter membrane time constant in F mice. (1-way ANOVAs, P2: F(3,59) = 1.175, p =0.327; P4-6: F(3,66) =0.934, p =0.430; P8-10: F(3,81) = 3.405, p =0.024; P14: F(3,73) = 2.283, p = 0.093. Data are presented as means (bars), error bars are standard error of the mean (SEM), dots are individual neurons from at least 3 animals per group. *p<0.05, control male vs. ethanol male; ^@@^p<0.01, control female vs. ethanol male. Download Figure 4-1, TIF file.

10.1523/ENEURO.0448-24.2025.f4-2 Figure 4-2 **Prenatal ethanol exposure does not alter membrane capacitance of developing striatal GABAergic interneurons (GINs) or striatal projection neurons (SPNs)** **(A)** Membrane capacitance was unaffected by prenatal ethanol exposure in striatal GINs or **(B)** SPNs. Data are presented as means (bars), error bars are standard error of the mean (SEM), dots are individual neurons from at least 3 animals per group. Download Figure 4-2, TIF file.

SPNs also significantly differed in AP threshold following prenatal ethanol exposure depending on postnatal age, with a trend toward differences in the effects of prenatal ethanol exposure on APs recorded from SPNs from male and female animals depending on the postnatal day (three-way ANOVA, exposure × sex × postnatal day: *p* = 0.051, exposure × postnatal day: *p* < 0.001; Table 4, Fig. 4*C*). At P2 prenatal ethanol exposure resulted in depolarized AP firing threshold in SPNs from male mice relative to those from control-fed male mice, while SPN AP threshold was unaffected in female mice (Fig. 4*C*). At P14, prenatal ethanol exposure resulted in a hyperpolarized AP threshold in both male and female mice relative their control-fed male and female counterparts (Fig. 4*C*). The half-width of APs recorded from SPNs was also affected by prenatal ethanol exposure (three-way ANOVA, exposure: *p* < 0.001; Fig. 4*D*, Table 4). SPN half-widths tended to be increased in mice exposed prenatally to ethanol; however, no significant differences in AP half-width were observed between groups on any postnatal day (Fig. 4*D*, Table 4). As observed in striatal GINs, AP peak amplitude was also unaltered by prenatal ethanol exposure regardless of postnatal day in SPNs from male and female mice (Table 4, Extended Data Fig. 3-1*B*).

**Table 4.** Striatal GIN and SPN action potential (AP) characteristic statistics

Effects	GIN AP threshold	GIN AP half-width	GIN AP peak amplitude	SPN AP threshold	SPN AP half-width	SPN AP peak amplitude
Exposure (E)	*F*_(1,231)_ = 6.504, *p = 0.011	*F*_(1,230)_ = 7.020, *p = 0.019	*F*_(1,231)_ = 0.646, *p* = 0.422	*F*_(1,271)_ = 0.034, *p* = 0.853	*F*_(1,271)_ = 13.193, ***p < 0.001	*F*_(1,271)_ = 0.006, *p* = 0.939
Sex (S)	*F*_(1,231)_ = 10.088, **p = 0.002	*F*_(1,230)_ = 7.268, *p = 0.017	*F*_(1,231)_ = 0.542, *p* = 0.463	*F*_(1,271)_ = 0.016, *p* = 0.899	*F*_(1,271)_ = 0.035, *p* = 0.852	*F*_(1,271)_ = 0.397, *p* = 0.529
Postnatal day (P)	*F*_(3,231)_ = 12.869, **p < 0.001	*F*_(3,230)_ = 81.193, ***p < 0.001	*F*_(3,231)_ = 2.121, *p* = 0.098	*F*_(3,271)_ = 3.173 *p = 0.025	*F*_(3,271)_ = 13.813, ***p < 0.001	*F*_(3,271)_ = 13.990, ***p < 0.001
E × S	*F*_(1,231)_ = 0.976, *p* = 0.324	*F*_(1,230)_ = 12.062, ***p < 0.001	*F*_(1,231)_ = 0.021, *p* = 0.884	*F*_(1,271)_ = 1.698, *p* = 0.194	*F*_(1,271)_ = 1.165, *p* = 0.281	*F*_(1,271)_ = 0.980, *p* = 0.323
E × P	*F*_(3,231)_ = 3.178, *p = 0.025	*F*_(3,230)_ = 1.977, *p* = 0.118	*F*_(3,231)_ = 1.405, *p* = 0.242	*F*_(3,271)_ = 7.306, ***p < 0.001	*F*_(3,271)_ = 2.193, *p* = 0.089	*F*_(3,271)_ = 1.544, *p* = 0.203
P × S	*F*_(3,231)_ = 5.538, **p = 0.001	*F*_(3,230)_ = 5.826, ***p < 0.001	*F*_(3,231)_ = 0.300, *p* = 0.826	*F*_(3,271)_ = 0.454, *p* = 0.715	*F*_(3,271)_ = 0.326, *p* = 0.807	*F*_(3,271)_ = 1.424, *p* = 0.236
E × S × P	*F*_(3,231)_ = 3.316, *p = 0.021	*F*_(3,230)_ = 7.781, ***p < 0.001	*F*_(3,231)_ = 1.197, *p* = 0.312	*F*_(3,271)_ = 2.620, *p* = 0.051	*F*_(3,230)_ = 0.020, *p* = 0.996	*F*_(3,230)_ = 0.349, *p* = 0.790

Statistical differences in AP threshold, half-width, and peak amplitude were determined with three-way ANOVAs. Major effects: exposure (control chow vs prenatal ethanol exposure embryonic day 13.5–16.5), sex (male vs female), and postnatal day (P2, P4, P6, P8, P10, and P14), with interactions: E × S (exposure × sex), E × P (exposure × postnatal day), P × S (postnatal day × sex), and E × S × P (exposure × sex postnatal day). Statistics with *p* value ≤0.05 are bolded.

Taken together, these data indicate differences in the effects of prenatal ethanol exposure on the development of SPNs in female and mice. While SPNs from both male and female mice displayed increased AP half-widths and firing rates during early postnatal development following prenatal ethanol exposure, increases in SPN AP firing rate occur later (P8–10) in male mice relative to female mice (P4–6; Fig. 4*A*,*D*). Consistent with what we observed in striatal GINs, SPNs from male mice also displayed more depolarized AP thresholds following prenatal ethanol exposure at P2 (Fig. 4*C*). Alternatively, by the end of the second postnatal week, SPNs from both displayed more hyperpolarized AP thresholds (Fig. 4*C*). This shift in AP threshold coincides with increased firing rates in response to lower current inputs in SPNs from female mice following prenatal ethanol exposure, while SPNs from male mice displayed decreased firing rates regardless of the degree of depolarizing current input (Fig. 4*A*).

#### Striatal GINs: intrinsic electrical properties

Given the significant differences in the firing properties of striatal GINs following prenatal ethanol exposure, we next asked if RMP, IR, membrane capacitance, and membrane time constant of developing striatal GINs and SPNs were also changed. Prenatal ethanol exposure significantly altered the RMP of striatal GINs, in sex- and postnatal day-dependent ways (three-way ANOVA, exposure: *p* = 0.055, sex: *p* = 0.027; exposure × sex × postnatal day: *p* = 0.035, exposure × sex: *p* = 0.019, exposure × postnatal day: *p* = 0.013, sex × postnatal day: *p* < 0.001; Table 5, Extended Data Fig. 3-1*B*). However, no significant differences between groups were observed on individual postnatal days (Extended Data Fig. 3-1*B*). IR, membrane time constant, and capacitance of striatal GINs were unaltered by prenatal ethanol exposure (Table 3; Extended Data Figs. 3-1*C,D*, 3-2*A*).

**Table 5.** Striatal GIN and SPN membrane properties statistics

Effects	GIN RMP	GIN input resistance	GIN membrane time constant	GIN capacitance	SPN RMP	SPN input resistance	SPN membrane time constant	SPN capacitance
Exposure (E)	*F*_(1,230)_ = 3.728, *p* = 0.055	*F*_(1,232)_ = 1.254, *p* = 0.264	*F*_(1,197)_ = 0.249, *p* = 0.619	*F*_(1,197)_ = 0.642, *p* = 0.424	*F*_(1,271)_ = 10.503, **p = 0.001	*F*_(1,271)_ = 8.100, **p = 0.005	*F*_(1,204)_ = 5.814, *p = 0.017	*F*_(1,204)_ = 2.525, *p* = 0.114
Sex (S)	*F*_(1,230)_ = 4.940, *p = 0.027;	*F*_(1,232)_ = 0.530, *p* = 0.467	*F*_(1,197)_ = 0.718, *p* = 0.398	*F*_(1,197)_ = 0.187, *p* = 0.666	*F*_(1,271)_ = 0.831, *p* = 0.363	*F*_(1,271)_ = 0.009, *p* = 0.923	*F*_(1,204)_ = 0.017, *p* = 0.897	*F*_(1,204)_ = 0.020, *p* = 0.888
Postnatal day (P)	*F*_(3,230)_ = 22.167, ***p < 0.001	*F*_(3,232)_ = 95.984 ***p < 0.001	*F*_(3,197)_ = 11.167, ***p < 0.001	*F*_(3,197)_ = 14.176, ***p < 0.001	*F*_(3,271)_ = 4.910, **p = 0.002	*F*_(3,271)_ = 216.643, ***p < 0.001	*F*_(3,204)_ = 49.272, ***p < 0.001	*F*_(3,204)_ = 85.567, **p < 0.001
E × S	*F*_(1,230)_ = 6.523, *p = 0.019	*F*_(1,232)_ = 1.404, *p* = 0.237	*F*_(1,197)_ = 0.257, *p* = 0.613,	*F*_(1,197)_ = 0.646, *p* = 0.422	*F*_(1,271)_ = 1.418, *p* = 0.235	*F*_(1,271)_ = 0.484, *p* = 0.487	*F*_(1,204)_ = 0.086, *p* = 0.770	*F*_(1,204)_ = 2.706, *p* = 0.102
E × P	*F*_(3,230)_ = 3.671, *p = 0.013	*F*_(3,232)_ = 0.791, *p* = 0.500	*F*_(3,197)_ = 0.362, *p* = 0.781	*F*_(3,197)_ = 0.463, *p* = 0.708	*F*_(3,271)_ = 1.295, *p* = 0.276	*F*_(3,271)_ = 1.555, *p* = 0.201	*F*_(3,204)_ = 0.937, *p* = 0.424	*F*_(3,204)_ = 0.680, *p* = 0.565
P × S	*F*_(3,230)_ = 4.065, ***p < 0.001	*F*_(3,232)_ = 1.378, *p* = 0.250	*F*_(3,197)_ = 0.412, *p* = 0.745	*F*_(3,197)_ = 1.950, *p* = 0.123	*F*_(3,271)_ = 1.339, *p* = 0.262	*F*_(3,271)_ = 0.369, *p* = 0.775	*F*_(3,204)_ = 4.081, **p = 0.008	*F*_(3,204)_ = 0.284, *p* = 0.837
E × S × P	*F*_(3,230)_ = 2.917, *p = 0.035	*F*_(3,232)_ = 1.083, *p* = 0.357,	*F*_(3,197)_ = 2.120, *p* = 0.099	*F*_(3,197)_ = 1.440, *p* = 0.232	*F*_(3,271)_ = 0.670, *p* = 0.571	*F*_(3,271)_ = 0.038, *p* = 0.990	*F*_(3,204)_ = 0.815, *p* = 0.487	*F*_(3,204)_ = 1.645, *p* = 0.180

Statistical differences in resting membrane potential (RMP), input resistance, membrane time constant, and capacitance were determined with three-way ANOVAs. Major effects: exposure (control chow vs prenatal ethanol exposure embryonic day 13.5–16.5), sex (male vs female), and postnatal day (P2, P4, P6, P8, P10 and P14), with interactions: E × S (exposure × sex), E × P (exposure × postnatal day), P × S (postnatal day × sex), and E × S × P (exposure × sex postnatal day). Statistics with *p* value ≤0.05 are bolded.

These data indicate that increases in striatal GIN AP firing rates following prenatal ethanol exposure (Fig. 3*A*,*C*) depend on the postnatal age and occur in the absence of obvious differences in other intrinsic electrical properties of striatal GINs (Extended Data Fig. 4-1*B*).

#### Striatal SPNs: intrinsic electrical properties

Prenatal ethanol exposure resulted in significant differences in the RMP of developing SPNs in both male and female mice (three-way ANOVA, exposure: *p* = 0.001; Table 3, Extended Data Fig. 4-1*B*). At P4–6 prenatal ethanol exposure resulted in more depolarized RMP in SPNs in male mice relative to control-fed male and female mice but did not result in statistically significant differences in SPNs from female mice (Extended Data Fig. 4-1*B*). A similar trend was observed at P2, though differences were not significant (one-way ANOVA, *p* = 0.070). Significant differences in SPN RMP were not detected during the second postnatal week (Extended Data Fig. 4-1*B*). Prenatal ethanol exposure also resulted in more depolarized RMP in SPNs from female mice relative to those in SPNs from control-fed female mice at both P2 and P4–6, though these differences did not reach statistical significance (Extended Data Fig. 4-1*B*).

Unlike striatal GINs, the IR of SPNs was significantly affected by prenatal ethanol exposure, independent of sex or postnatal day (three-way ANOVA: main effects: exposure: *p* = 0.005; Table 3). Though significant differences were not observed on individual postnatal days, we observed a trend toward an increase in IR of SPN at P2 and P4–6 in SPNs from both male and female mice relative those fed a control diet (Extended Data Fig. 4-1*C*). Membrane time constant of SPNs also significantly differed following a prenatal ethanol exposure as well as between male and female mice depending upon the postnatal day (three-way ANOVA: exposure: *p* = 0.017, sex × postnatal day: *p* = 0.008; Table 5). On P8–10, SPNs recorded from control-fed males displayed a significantly increased membrane time constant relative to those from ethanol-exposed male mice (Extended Data Fig. 4-1*D*). The membrane capacitance of SPNs was unaffected by prenatal ethanol exposure and did not differ between sexes (Extended Data Fig. 3-2*B*, Table 5).

Consistent with observed increases in SPN AP half-width during early postnatal development (P2–6), the more depolarized RMP and increased IR of developing SPNs suggest that prenatal ethanol exposure results in a delayed functional maturation of SPNs in mice of both sexes, although these effects tended to be more pronounced in SPNs from male mice. These data also suggest that alterations to the intrinsic properties precede increases in SPN firing rate observed following prenatal ethanol exposure in SPNs from female mice (P4–6) and male mice (P8–10; Extended Data Fig. 4-1*A*).

### Prenatal ethanol exposure alters the development of glutamatergic and GABAergic synaptic connections to striatal GINs and SPNs in a sex-dependent manner during the first two postnatal weeks

The intrinsic excitability and firing properties of striatal neurons is modified by synaptic input in both developing and adult animals (Wilson and Kawaguchi, 1996; Lieberman et al., 2018; Lahiri and Bevan, 2020). Thus, we asked how differences in the functional properties might relate to the development of glutamatergic and synaptic afferents of striatal GINs and SPNs. Striatal GINs and SPNs receive convergent glutamatergic input from the cortex, thalamus, and limbic regions including the hippocampus and amygdala and share local GABAergic inputs from GINs and SPNs, as well as distant inhibitory inputs from cortex and pallidum (Tepper et al., 2008; Wall et al., 2013; Melzer et al., 2017; Klug et al., 2018; Bertero et al., 2020). Glutamatergic and GABAergic synaptic currents have been observed in embryonic SPNs and gradually increase over the first two postnatal weeks coinciding with the onset of mature functional properties in SPNs (Tepper et al., 1998; Dehorter et al., 2011; Kozorovitskiy et al., 2012; Sohur et al., 2014; Peixoto et al., 2016; Krajeski et al., 2019). When striatal GINs first receive input from synaptic afferents is not yet known.

We performed whole-cell patch-clamp recordings using acute coronal sections from behaviorally tested neonates to record GABAergic and glutamatergic sPSCs from developing striatal GINs and SPNs. We asked if prenatal ethanol exposure altered the frequency and amplitude of glutamatergic sPSCs during the first two postnatal weeks.

#### Striatal GINs: glutamatergic sPSCs

**Figure 5.**
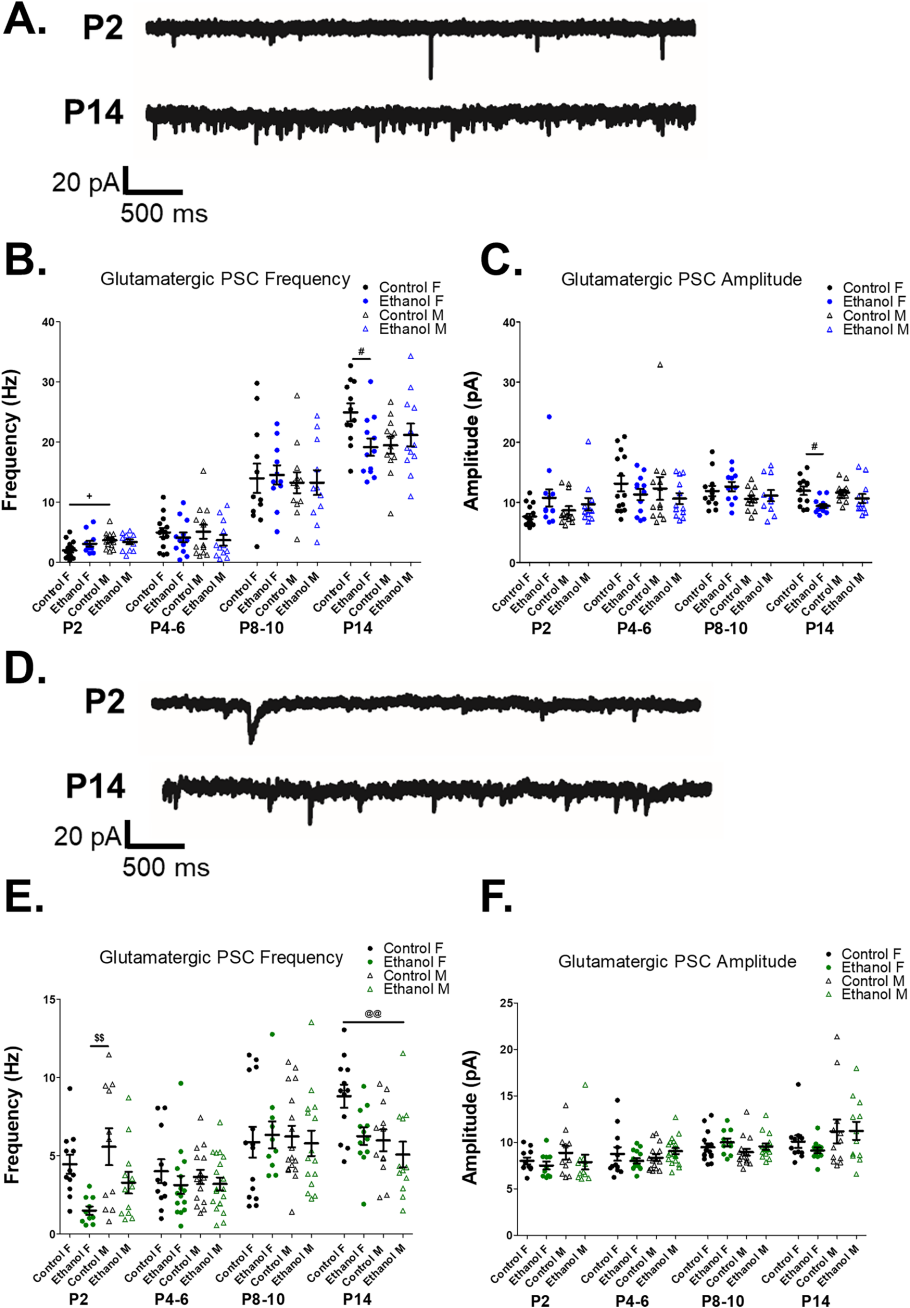
Glutamatergic synaptic activity in the developing striatum is disrupted by prenatal ethanol exposure depending on sex and neuronal subtype: striatal GABAergic interneurons (GINs) and striatal projection neurons (SPNs). ***A***, Example whole-cell voltage-clamp recordings of spontaneous glutamatergic postsynaptic current (sPSC) recordings from striatal GINs in control-fed female mice at P2 and P14. ***B***, Prenatal ethanol exposure differentially effects the frequency of glutamatergic sPSCs recorded in striatal GIN from female and male mice depending upon their postnatal age. At P2, control-fed male mice demonstrate a higher glutamatergic sPSC frequency than control-fed female mice, while frequency does not differ in GINs from ethanol-exposed male versus female mice (1-way ANOVA, *F*_(3,49)_ = 3.884, *p *= 0.014; Bonferroni’s post hoc tests: control M vs control F: *p* = 0.017). At P14, prenatal ethanol exposure results in a decreased frequency of glutamatergic sPSCs in female mice relative to control-fed female mice (1-way ANOVA, *F*_(3,47)_ = 4.007, *p* = 0.025, Bonferroni’s post hoc tests: ethanol F vs control F: *p* = 0.037). Prenatal ethanol exposure does not affect glutamatergic sPSC frequency in striatal GINs from P4–6 or P8–10 mice (1-way ANOVAs, P4–6: *F*_(3,47)_ = 0.530, *p* = 0.664; P8–10: *F*_(3,47)_ = 0.092, *p* = 0.964). ***C***, Prenatal ethanol exposure decreases glutamatergic sPSC amplitude in striatal GINs from P14 female mice relative to control-fed female mice but does not alter glutamatergic sPSC frequency in striatal GINs from female between P2 and P10 or from male mice (1-way ANOVAs, P2: *F*_(3,49)_ = 2.283, *p* = 0.091; P4–6: *F*_(3,47)_ = 0.666, *p* = 0.577; P8–10: *F*_(3,45)_ = 1.266, *p* = 0.298; P14: *F*_(3,47)_ = 0.666, *p* = 0.012, Bonferroni’s post hoc tests: ethanol F vs control: *p* = 0.012). ***D***, Example glutamatergic sPSC recordings from SPNs in control-fed female mice at P2 and P14. ***E***, Prenatal ethanol exposure results in an early postnatal (P2) decrease in glutamatergic sPSC frequency in SPNs from female mice, relative to control-fed male mice (1-way ANOVA, *F*_(3,41)_ = 4.852, *p *= 0.006; Bonferroni’s post hoc tests: control M vs ethanol F: *p* = 0.005). At P14, prenatal ethanol exposure resulted in significantly decreased glutamatergic sPSC frequency in SPNs from male mice relative to control-fed female mice (1-way ANOVA, *F*_(3,47)_ = 4.853, *p *= 0.005, Bonferroni’s post hoc tests: control F vs ethanol M: *p* = 0.005). Prenatal ethanol exposure did not affect glutamatergic sPSC frequency in SPNs from male and female mice at P4–6 or P8–10 (1-way ANOVAs, P4–6: *F*_(3,56)_ = 0.537, *p* = 0.659; P8–10: *F*_(3,52)_ = 0.094, *p* = 0.963). ***F***, Prenatal ethanol exposure did not alter amplitude of glutamatergic sPSCs recorded from SPN during the first two postnatal weeks (one-way ANOVAs, P2: *F*_(3,41)_ = 0.861, *p *= 0.469; P4–6: *F*_(3,56)_ = 1.332, *p* = 0.273; P8–10: *F*_(3,52)_ = 1.341, *p* = 0.271; P14: *F*_(3,47)_ = 1.093, *p *= 0.362). Data are presented as means (bars), error bars are standard error of the mean (SEM), dots are individual neurons from at least three animals per group. For all: ^+^*p* < 0.05, control M versus control F; ^#^*p* < 0.05, control F versus ethanol F; ^@@^*p* < 0.01, control F versus ethanol M; ^$$^*p* < 0.01, control M versus ethanol F.

Though we did not identify significant effects of prenatal ethanol exposure or sex on glutamatergic sPSC frequency in striatal GINs, there emerged a trend toward an exposure × sex × postnatal day interaction suggesting that the prenatal ethanol exposure alters the frequency of glutamatergic sPSCs in striatal GINs in a sex-dependent manner that varies across early postnatal development (three-way ANOVA, exposure × sex × postnatal day: *p* = 0.058; Table 6, Fig. 5*C*). We then compared glutamatergic sPSC frequency in striatal GINs from control female, ethanol-exposed female, control-fed male, and ethanol-exposed male mice by postnatal day (P2, P4–6, P8–10, and P14). At P2, prenatal ethanol exposure increased glutamatergic sPSC frequency in GINs from control-fed male mice relative to those from control-fed female mice, though no differences were observed between ethanol-exposed male and female mice (Fig. 5*C*). At P14, prenatal ethanol exposure decreased the frequency of glutamatergic PSCs in female mice relative to control female mice (Fig. 5*C*).

**Table 6.** Striatal GIN and SPN spontaneous postsynaptic current (sPSC) statistics

Effects	GIN glutamatergic sPSC frequency	GIN glutamatergic sPSC amplitude	SPN glutamatergic sPSC frequency	SPN glutamatergic sPSC amplitude	GIN GABAergic sPSC frequency	GIN GABAergic sPSC amplitude	SPN GABAergic sPSC frequency	SPN GABAergic sPSC amplitude
Exposure (E)	*F*_(1,182)_ = 0.883, *p* = 0.349	*F*_(1,182)_ = 0.164, *p* = 0.686	*F*_(1,193)_ = 12.003, ***p < 0.001	*F*_(1,193)_ = 0.253, *p* = 0.615	*F*_(1,182)_ = 2.973, *p* = 0.476	*F*_(1,172)_ = 0.751, *p* = 0.387	*F*_(1,193)_ = 9.687, **p = 0.002	*F*_(1,192)_ = 0.009, *p* = 0.924
Sex (S)	*F*_(1,182)_ = 0.883, *p* = 0.473	*F*_(1,182)_ = 0.770, *p* = 0.381	*F*_(1,193)_ = 0.305, *p* = 0.581	*F*_(1,193)_ = 3.265, *p* = 0.072	*F*_(1,182)_ = 6.748, *p = 0.010	*F*_(1,172)_ = 0.851, *p* = 0.358	*F*_(1,193)_ = 0.009, *p* = 0.923	*F*_(1,192)_ = 7.388, **p = 0.007
Postnatal age (P)	*F*_(1,182)_ = 168.705, ***p < 0.001	*F*_(1,182)_ = 6.263, ***p < 0.001	*F*_(1,193)_ = 18.882, ***p < 0.001	*F*_(1,193)_ = 12.194, ***p < 0.001	*F*_(3,182)_ = 88.952, ***p < 0.001	*F*_(1,172)_ = 9.632, ***p < 0.001	*F*_(1,193)_ = 38.104, ***p < 0.001	*F*_(1,192)_ = 9.302, ***p < 0.001
E × S	*F*_(1,182)_ = 0.909, *p* = 0.342	*F*_(1,182)_ = 0.026, *p* = 0.872	*F*_(1,193)_ = 2.621, *p* = 0.528	*F*_(1,193)_ = 0.946, *p* = 0.332	*F*_(1,182)_ = 0.064, *p* = 0.801	*F*_(1,172)_ = 0.311, *p* = 0.578	*F*_(1,193)_ = 0.105, *p* = 0.747	*F*_(1,192)_ = 0.276, *p* = 0.600
E × P	*F*_(3,182)_ = 0.789, *p* = 0.501	*F*_(3,182)_ = 3.885, *p = 0. 010	***F*_(3,193)_ = 2.621, *p* = 0.052**	*F*_(3,193)_ = 1.199, *p* = 0.311	*F*_(3,182)_ = 1.859, *p* = 0.138	*F*_(3,172)_ = 4.467, **p = 0.005,	*F*_(3,193)_ = 0.365, *p* = 0.778	*F*_(3,192)_ = 2.836, *p = 0.039,
P × S	*F*_(3,182)_ = 0.807, *p* = 0.492	*F*_(3,182)_ = 0.692, *p* = 0.058	***F*_(3,193)_ = 3.487, *p* = 0.017**	*F*_(3,193)_ = 1.805, *p* = 0.148	*F*_(3,182)_ = 2.581, *p* = 0.055	*F*_(3,172)_ = 0.775, *p* = 0.509	*F*_(3,193)_ = 2.107, *p* = 0.101	*F*_(3,192)_ = 1.155, *p* = 0.328
E × S × P	*F*_(3,182)_ = 2.536, *p* = 0.058	*F*_(3,182)_ = 0.603, *p* = 0.614	*F*_(3,193)_ = 0.526, *p* = 0.665	*F*_(3,193)_ = 0.706, *p* = 0.549	*F*_(3,182)_ = 0.171, *p* = 0.916	*F*_(3,172)_ = 1.638, *p* = 0.182	*F*_(3,193)_ = 2.099, *p* = 0.102	*F*_(3,192)_ = 0.324, *p* = 0.808

Statistical differences in glutamatergic and GABAergic postsynaptic current frequency and amplitude were determined with three-way ANOVAs. Major effects: exposure (control chow vs prenatal ethanol exposure embryonic day 13.5–16.5), sex (male vs female), and postnatal day (P2, P4, P6, P8, P10, and P14), with interactions: E × S (exposure × sex), E × P (exposure × postnatal day), P × S (postnatal day × sex), and E × S × P (exposure × sex postnatal day). Statistics with *p* value ≤0.05 are bolded.

Depending on the postnatal day assessed, prenatal ethanol exposure differentially altered the amplitude of glutamatergic sPSCs recorded from striatal GINs (three-way ANOVA, exposure × postnatal day: *p* = 0.010; Table 6, Fig. 5*D*). At P14, prenatal ethanol exposure resulted in decreased glutamatergic sPSC amplitude in striatal GINs from female mice relative to those from control-fed female mice (Fig. 5*D*).

#### SPNs: glutamatergic sPSCs

Prenatal ethanol exposure significantly changed the frequency of glutamatergic sPSCs recorded from SPNs, revealing a trend toward an exposure × sex × postnatal day interaction and suggesting that similar to GINs, prenatal ethanol exposure alters the frequency of glutamatergic sPSCs in SPNs in a sex-dependent manner that varies across early postnatal development (three-way ANOVA, exposure: *p* < 0.001, exposure × postnatal day: *p* = 0.052, sex × postnatal day: *p* = 0.017; Table 6). We determined that at P2 prenatal ethanol exposure resulted in a decrease in the frequency of glutamatergic sPSCs in female mice, relative to control-fed male mice, and observed a trend toward decreased frequency relative to control-fed female mice (*p* = 0.061; Fig. 5*E*). At P14, significant differences in ethanol-exposed male and control-fed female mice were evident (Fig. 5*E*). Prenatal ethanol exposure did not alter the amplitude of glutamatergic sPSCs recorded from SPNs, independent of sex and postnatal age (Table 6, Fig. 5*F*).

Overall, in striatal GINs of female mice, prenatal ethanol exposure results in an early postnatal (P2) increase in the frequency of glutamatergic sPSC suggesting an increase in glutamatergic synaptic inputs to striatal GINs coinciding with a decrease in glutamatergic inputs to SPNs. These differences resolved by the end of the first postnatal week. However, they are replaced by P14 with decreases in both sPSC frequency of striatal GINs suggesting that prenatal ethanol exposure results later in diminished glutamatergic neurotransmission via both pre- and postsynaptic mechanisms (Fig. 5*B*,*C*,*E*,*F*). In contrast, though prenatal ethanol exposure also resulted in an early postnatal decrease glutamatergic sPSC frequency in SPNs from male mice, this difference was not statistically significant and does not coincide with an increased glutamatergic sPSC frequency in striatal GINs in male mice (Fig. 5*B*,*C*,*E*,*F*). Following prenatal ethanol exposure, striatal GINs from male mice also failed to display decreases in glutamatergic sPSC frequency and amplitude observed in GINs from female mice at P14 but unlike female mice, develop a more pronounced decrease in SPN glutamatergic sPSC frequency by P14.

#### Striatal GINs: GABAergic sPSCs

**Figure 6.**
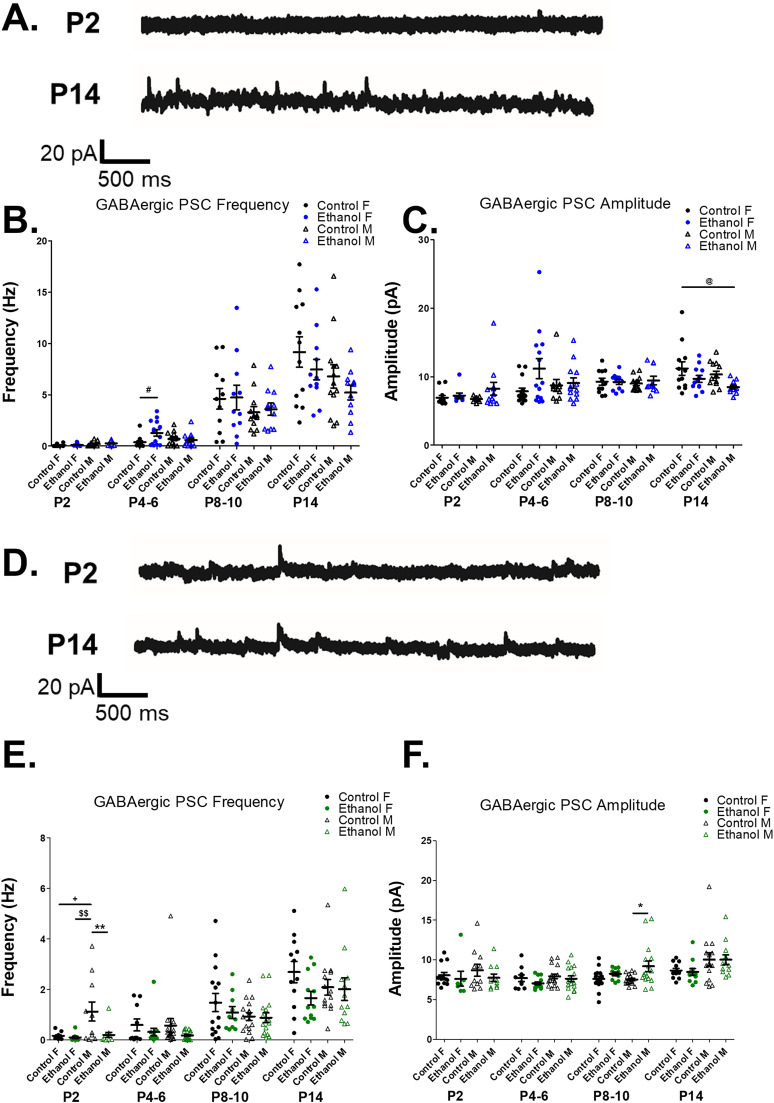
GABAergic synaptic activity in the developing striatum is disrupted by prenatal ethanol exposure depending on sex and neuronal subtype: striatal GABAergic interneurons (GINs) and striatal projection neurons (SPNs). ***A***, Example whole-cell voltage-clamp recordings of spontaneous GABAergic postsynaptic current (sPSC) recordings from striatal GINs in control-fed female mice at P2 and P14. ***B***, Prenatal ethanol exposure increases the frequency of GABAergic sPSC recorded from striatal GINs in female mice relative to control-fed F mice at P4–6 but does not alter GABAergic sPSC frequency in F mice at P2, P8–10, or P14 and does not affect GABAergic sPSC frequency in male mice (1-way ANOVAs, P2: *F*_(3,48)_ = 2.919, *p* 0.043; P4–6: *F*_(3,52)_ = 3.307, *p* = 0.028; Bonferroni’s post hoc tests: control F vs ethanol F: *p* = 0.023; P8–10: *F*_(3,41)_ = 0.699, *p* 0.558; P14: *F*_(3,47)_ = 2.005, *p* 0.127). ***C***, Striatal GINs from control-fed F mice had significantly increased GABAergic sPSC amplitude relative to those from control-fed M and ethanol-exposed M P14 mice (1-way ANOVA, *F*_(3,47)_ = 3.150, *p =* 0.034, Bonferroni’s post hoc tests: control M vs control F: *p* = 0.028, control F vs ethanol M: *p* = 0.028). Prenatal ethanol exposure did not alter GABAergic sPSC amplitude between P2 and P10 (one-way ANOVAs, P2: *F*_(3,40)_ = 1.461, *p* = 0.240; P4–6: *F*_(3,52)_ = 2.253, *p =* 0.094; P8–10: *F*_(3,41)_ = 0.160, *p* = 0.923, P14: *F*_(3,44)_ = 3.150, *p* = 0.034, Bonferroni’s post hoc tests: ethanol M versus control F: *p* = 0.028. ***D***, Example whole-cell voltage-clamp recordings of spontaneous GABAergic sPSC recordings from SPNs in control-fed female mice at P2 and P14. ***E***, Prenatal ethanol exposure significantly decreased the frequency of GABAergic sPSC in SPNs from M mice at P2, relative to control-fed M and F mice, and ethanol-exposed F mice (1-way ANOVA, *F*_(3,42)_ = 6.383, *p* = 0.001; Bonferroni’s post hoc test: control M vs ethanol M: *p* = 0.008, control M vs control F: *p* = 0.004, control M vs ethanol F: *p* = 0.004). Prenatal ethanol exposure did not alter GABAergic sIPSC frequency in SPNs P4–14 (1-way ANOVAs, P4–6: *F*_(3,57)_ = 0.921, *p* = 0.437; P8–10: *F*_(3,54)_ = 0.921, *p* = 0.437; P14: *F*_(3,46)_ = 1.344, *p* = 0.272). ***F***, At P8–10, prenatal ethanol exposure increases the amplitude of GABAergic SPCs in SPNs from M mice relative to control-fed M (1-way ANOVA, *F*_(3,54)_ = 3.623, *p* = 0.019, Bonferroni’s post hoc: control M vs ethanol M: *p* = 0.027, control F vs ethanol M: *p* = 0.052). Prenatal ethanol exposure does not affect GABAergic sPSC amplitude at P2, P4–6, or P14 (1-way ANOVAs, P2: *F*_(3,38)_ = 0.587, *p* = 0.627; P4–6: *F*_(3,57)_ = 1.920, *p* = 0.137, P14: *F*_(3,46)_ = 1.865, *p* = 0.149). Data are presented as means (bars), error bars are standard error of the mean (SEM), and dots are individual neurons from at least three animals per group. For all: *p < 0.05, **p < 0.01, control male versus ethanol male, ^+^*p* < 0.05, control M versus control F; ^#^*p* < 0.05, control F versus ethanol F; ^@^*p* < 0.05, control F versus ethanol M; ^$$^*p* < 0.01 control M versus ethanol F.

We next asked if the differences we found in glutamatergic sPSC frequency and amplitude might coincide with changes in GABAergic sPSCs following prenatal ethanol exposure. Although we identified a significant effect of biological sex on the frequency of GABAergic sPSCs in striatal GINs, we did not identify a significant effect of prenatal ethanol exposure (three-way ANOVA, sex: *p* = 0.010; Table 6, Fig. 6*C*). Despite the lack of an overall effect of prenatal ethanol exposure on GABAergic sPSC frequency in striatal GINs, we did uncover group-dependent differences in GABAergic sPSC frequency at P14, striatal GINs from control-fed female mice had a significantly increased GABAergic sPSC frequency relative to control-fed and ethanol-exposed male mice, while striatal GINs from ethanol-exposed female mice did not display a similar increase in frequency (Fig. 6*C*).

Alternatively, the amplitude of GABAergic sPSCs monitored in striatal GINs was significantly altered by prenatal ethanol exposure, depending on the postnatal day assessed and irrespective of sex (three-way ANOVA, exposure × postnatal day: *p* = 0.0050; Fig. 6*D*, Table 6). At P14, striatal GINs from control-fed female mice displayed decreased GABAergic sPSC amplitude relative to ethanol-exposed male mice, while a comparable decrease in GABAergic sPSC amplitude was not observed in ethanol-fed female mice (Fig. 6*D*).

#### SPNs: GABAergic sPSCs

Prenatal ethanol exposure significantly altered the frequency of GABAergic sPSCs in SPNs from both male and female mice across the first two postnatal weeks (three-way ANOVA, exposure: *p* = 0.002; Table 6, Fig. 6*E*). At P2, SPNs from control-fed male mice displayed a significantly higher GABAergic sPSC frequency relative to all other groups, while SPNs in male mice exposed prenatally to ethanol did not display a similar increase in GABAergic sPSC frequency (Fig. 6*E*). Although no significant differences in SPN GABAergic sPSC frequency were observed in female or male mice from P4 to P14, SPNs from both male and female mice tended to display decreased GABAergic SPN frequency following prenatal ethanol exposure in both male and female mice during this time period (Fig. 6*E*).

Depending on the postnatal age, the amplitude of GABAergic sPSCs monitored in SPNs was also significantly altered by prenatal ethanol exposure (three-way ANOVA, exposure × postnatal day: *p* = 0.039; Fig. 6*F*, Table 6). At P8–10, prenatal ethanol exposure resulted in a decrease in SPN GABAergic sPSC amplitude in male mice, relative to control-fed male mice (Fig. 6*F*). No differences were observed in GABAergic sPSC amplitude in female mice at P8–10 (Fig. 6*F*).

In summary, prenatal ethanol exposure differentially affected GABAergic synaptic activity in striatal neurons of female and male mice during the first two postnatal weeks, including both pre- and postsynaptic GABAergic neurotransmission in striatal GINs and SPNs, with more pronounced differences observed in striatal neurons from male mice.

### Prenatal ethanol exposure results in transient alterations in SPN morphology in both female and male mice

**Figure 7.**
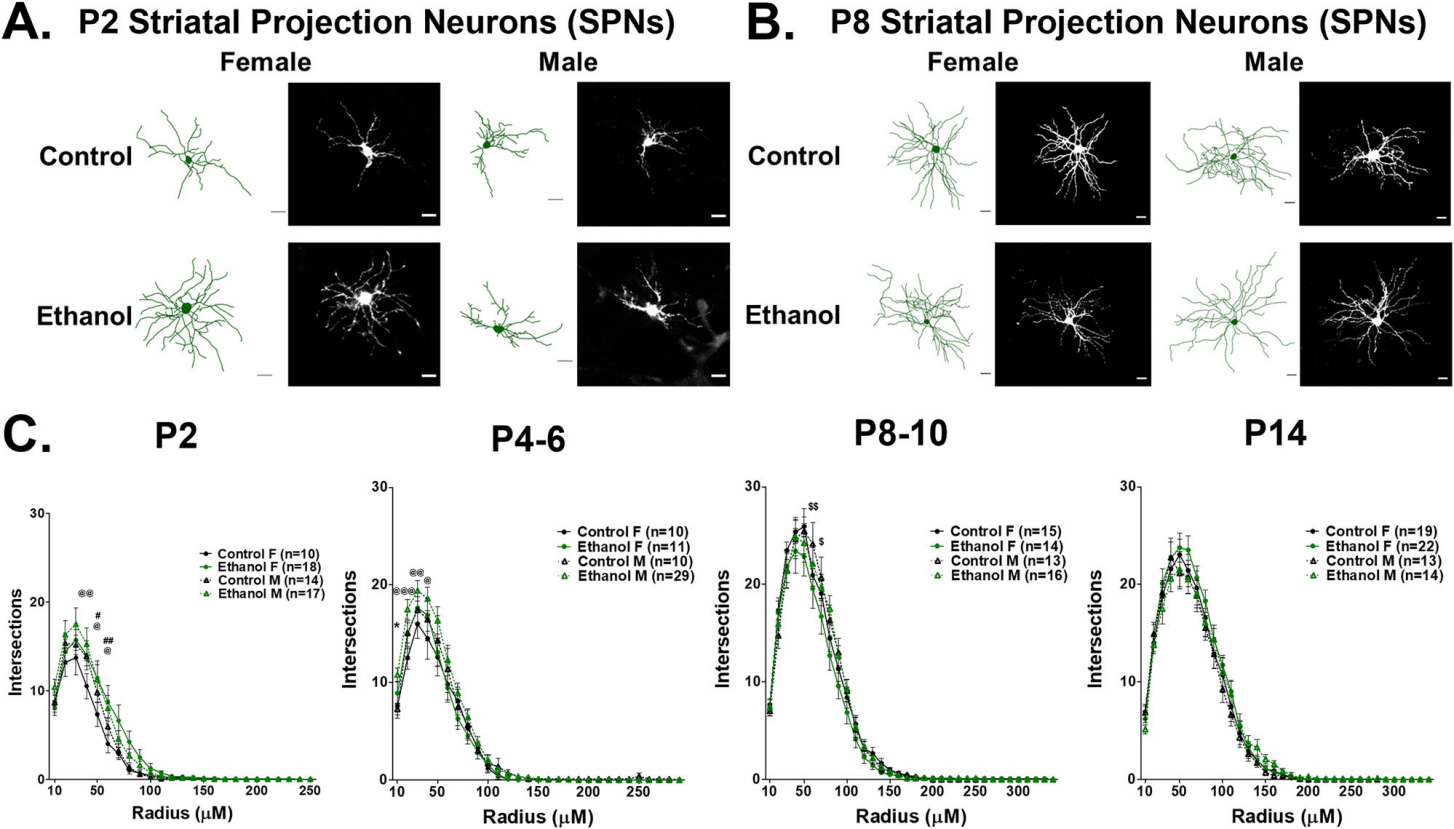
Prenatal ethanol exposure result in early postnatal increases in spiny projection neuron (SPN) dendritic morphology. ***A***, Representative reconstructions (right) and images (left) of neurobiotin-filled SPNs from female (F) control, male (M) control (top), F ethanol-exposed, and M ethanol-exposed (bottom) postnatal day (P) 2 neonates. ***B***, Representative reconstructions (right) and images (left) of neurobiotin-filled SPNs from F control, M control (top), F ethanol-exposed, and M ethanol-exposed (bottom) P8 neonates. ***C***, Sholl analysis indicates that prenatal ethanol exposure increases the dendritic complexity (number of intersections/increasing 10 µm radius) in a radius-dependent manner of SPNs from P2 F and M + mice relative to control-fed F mice, P4–6 M mice relative control-fed F mice, and decreased in P8–10 F mice relative to ethanol-exposed M and control-fed M, while differences have resolved by P14 (two-way repeated measures ANOVA, P2: group: *F*_(3,1320)_ = 1.559, *p* = 0.2097, radius: *F*_(24,1320)_ = 188.9, *p* < 0.001, group × radius: *F*_(72,1320)_ = 1.241, *p* = 0.0878, Bonferroni’s post hoc tests: ethanol F vs control F: radius = 50 µm, *t* = 3.348, *p* < 0.05, radius = 60 µm, *t* = 3.866, *p* < 0.01); ethanol M vs control F: radius = 40 µm, *t* = 3.837, *p* < 0.01, radius = 50 µm, *t* = 3.189, *p* < 0.05, radius = 60 µm, *t* = 3.241, *p* < 0.05; P4–6: group: *F*_(3,1596)_ = 1.701, *p* = 0.1770, radius: *F*_(28,1596)_ = 228.5, *p* < 0.001, group × radius: *F*_(84,1596)_ = 1.093, *p* = 0.2684, Bonferroni’s post hoc tests: ethanol M vs control F: radius = 20 µm, *t* = 4.540, *p* < 0.0001, 40 µm, *t* = 3.692, *p* < 0.01, 50 µm, *t* = 3.412, *p* < 0.05. P8–10: group: *F*_(3,1782)_ = 0.6876, *p* = 0.5635, radius: *F*_(33,1782)_ = 475.5, *p* < 0.0001, group × radius: *F*_(99,1782)_ = 0.8764, Bonferroni’s post hoc tests: ethanol F vs ethanol M: radius = 80 µm, *t* = 4.061, ethanol F vs control M: radius = 60 µM, *t* = 3.694, *p* < 0.01, 70 µm, *t* = 3.207, *p* < 0.05. P14: group: *F*_(3,2112)_ = 0.4037, *p* = 0.7508, radius *F*_(33,2112)_ = 437.2, *p* < 0.0001, group × radius: *F*_(99,2112)_ = 0.6128, *p* = 0.9990). For all images, scale bar, 20 µm. Data are presented as means (bars), error bars are standard error of the mean (SEM), and dots are individual neurons from at least three animals per group. For all: ^+^*p* < 0.05, ^++^*p* < 0.01, control M versus control F; ^#^*p* < 0.05, ^##^*p* < 0.01, control M versus ethanol M, ^@^*p* < 0.05; ^@@@^*p* < 0.001, control F versus ethanol M, ^$$^*p* < 0.01 control M versus ethanol F; ^x^*p* < 0.05, ^xx^*p* < 0.01, ethanol F versus ethanol M. Extended Data Figure 7-1.

Concurrent with rapid changes in functional properties, and synaptic inputs during the first two postnatal weeks, SPNs undergo considerable morphological maturation (Tepper et al., 1998; Dehorter et al., 2011; Peixoto et al., 2016; Krajeski et al., 2019). The dendritic complexity of SPNs has been closely associated with their excitability both during development and in adulthood, with less excitable SPN neurons observed to bear longer and more complex dendritic arbors (Gertler et al., 2008; Cazorla et al., 2014; Lieberman et al., 2018; Krajeski et al., 2019). Thus, we asked whether altered SPN morphology might accompany differences in the functional maturation and synaptic connectivity of SPNs. Spiny projection neurons were filled with a neurobiotin tracer during whole-cell patch-clamp electrophysiology experiments and then traced and SPN dendrites reconstructed for Sholl analysis (Fig. 7).

10.1523/ENEURO.0448-24.2025.f7-1 Figure 7-1 **Prenatal ethanol exposure result in early postnatal increases in spiny projection neuron (SPN) dendritic morphology: length, number, branching, and soma area. (A)** Prenatal ethanol exposure decreased the mean length/dendrite (µM) in SPN F mice relative to control-fed F while control-male mice also displayed significantly decreased mean length/dendrite relative to control fed F at P2, while prenatal ethanol exposure resulted in no significant differences at P4-6. At P8-10 prenatal ethanol exposure increased the mean length/dendrite in SPNs from M mice relative to ethanol-exposed and control-fed F mice, control-fed male mice also displayed significantly increased mean length/dendrite relative to control-fed F mice. At P14, SPNs from ethanol-exposed M mice relative displayed a decreased mean length/dendrite relative to ethanol-exposed and control-fed F mice. (P2: (Kruskall-Wallis test, H(3) = 13.49, p=0.0039, Dunn’s post-hoc tests: ethanol F vs. control F: p<0.05, ethanol F vs. control M, p>0.05; P4-6: Kruskal-Wallis test, H(3) = 6.905, p=0.0750; P8-10: one-way ANOVA, F(3,59) = 6.276, p = 0.001, Bonferroni post-hoc tests: ethanol M vs. ethanol F, t= 3.427, p<0.01, ethanol M vs. control F, t=3.710, p<0.01; P14: one-way ANOVA, F(3,64) = 3.962, p = 0.0118, Bonferroni post-hoc tests: ethanol M vs. ethanol F, t= 3.308, p<0.01). **(B)** Prenatal ethanol exposure results in a transient increase in the number of dendrites in SPNs from P2 and P4-6 F mice relative to those from control-fed F and ethanol-exposed M mice of the same ages, that resolves by P8-10 (Kruskal-Wallis tests, P2: H(3)= 12.832, p=0.005, Dunn’s post-hoc tests: ethanol F vs. control F: p< 0.05, ethanol F vs. ethanol M, p<0.05; P4-6: H(3) = 14.116, p=0.003, Dunn’s post-hoc tests: ethanol F vs. control F: p<0.01, ethanol F vs. ethanol M, p<0.01; P8-10: H(3)= 0.897, p=0.826; P14: H(3)= 0.747, p=0.862). **(C)** Prenatal ethanol exposure resulted in trend towards a decreased mean number of nodes/dendrite in SPNs from P4-6 F mice relative to control-fed F mice, and ethanol-exposed M mice. Control-fed F mice had increased mean nodes/dendrite relative to control-fed M mice at P2. No differences in mean nodes/dendrite mean nodes per dendrite were observed in SPNs at P8-10, or P14 (P2: one-way ANOVA, F(3,58)=4.596, p= 0.061, Bonferroni post-hoc tests, control F vs. control M, p<0.05; P4-6: Kruskal-Wallis test, H(3) = 8.619. p=0.0348; P8-10: one-way ANOVA: F(3,59) = 1.340, P14: F(3,67) = 0.6633). **(D)** Prenatal ethanol exposure increased the soma area (µM^2^) if SPNs from F mice, relative to those from control-fed F mice at P4. SPNs from control-fed F mice also had decreased soma area relative to those from control-fed male mice at P4. No differences in soma area were observed at P2, P8-10 or P14. P2: one way ANOVA, F(3,58) = 0.4028, p = 0.7515; P4: one-way ANOVA, F(3,60) = 5.002, p<0.0038, Bonferroni post-hoc tests: ethanol F vs. control F, t=3.121, p<0.05, control F vs. control M, t = 3.591, p<0.01; P8-10: one-way ANOVA, F(3,59) = 0.7238, p= 0.5420; P14: Kruskall-Wallis test, H(3) = 6.232. Data are presented as means (bars), error bars are standard error of the mean (SEM), dots are individual neurons from at least 3 animals per group. For all: ^+^p<0.05, ^++^p<0.01, control M vs. control F; ^#^p<0.05, ^##^p<0.01, control M vs. ethanol M, ^@^p<0.05; ^@@@^p<0.001, control F vs. ethanol M, ^$$^ p<0.01 control M vs. ethanol F; ^x^p<0.05, ^xx^p<0.01, ethanol F vs. ethanol M. Download Figure 7-1, TIF file.

At P2, SPNs from female mice exposed prenatally to ethanol had more complex dendrites as measured by Sholl analysis compared with control-fed female mice, while increases in the dendritic complexity of SPNs were less pronounced in ethanol-exposed male mice relative to SPNs from control-fed male mice (Fig. 7*C*). The increases in dendritic complexity in female mice diminished over time, with significant differences evident only at P4–6 between SPNs from ethanol-exposed female mice and those of control and ethanol-exposed male mice (Fig. 7*C*). Alternatively, at both P2 and P8–10, SPNs from ethanol-exposed male mice displayed increased complexity relative to those in control-fed female mice but not control-fed male mice while that of SPNs from control-fed male and female mice did not differ (Fig. 7*C*).

Differences in the dendritic complexity may result from changes in the number of dendrites, dendritic length, or dendritic branchpoints (number of nodes) and may coincide with changes in the growth of other neuronal compartments, including the soma. We found that prenatal ethanol exposure differentially altered the number of dendrites in SPNs of female and male mice depending on the postnatal day (ordinal logistic regression exposure: *p* = 0.003, exposure × sex × postnatal day: *p* = 0.015; Extended Data Fig. 7-1*B*, Table 7). An increase in dendritic number was observed in SPNs from ethanol-exposed female mice relative to SPNs from control-fed female and ethanol-exposed male mice at P2 and persisted to P4–6.

**Table 7.** SPN dendritic morphology and soma area statistics

Effects	Mean length/dendrite (µm)	Mean nodes/dendrite (µm)	Soma area (µm^2^)
Exposure (E)	*F*_(1,232)_ = 1.030, *p* = 0.311	*F*_(1,232)_ = 0.007, *p* = 0.933	*F*_(1,232)_ = 1.030, *p* = 0.412
Sex (S)	*F*_(1,232)_ = 3.724, *p* = 0.055	*F*_(1,232)_ = 1.362, *p* = 0.244	*F*_(1,232)_ = 0.141, *p* = 0.707
Postnatal age (P)	*F*_(1,232)_ = 29.118, ***p < 0.001	*F*_(1,232)_ = 7.700, ***p < 0.001	*F*_(3,232)_ = 3.699,*p = 0.012
E × S	*F*_(1,232)_ = 1.162, *p* = 0.282	*F*_(1,232)_ = 7.159, **p = 0.008	*F*_(1,232)_ = 10.014,** *p* = 0.002
E × P	*F*_(3,232)_ = 1.503, *p* = 0.214	*F*_(3,232)_ = 0.901, *p* = 0.442	*F*_(3,232)_ = 1.503, *p* = 0.214
P × S	*F*_(3,232)_ = 11.774, ***p < 0.001	*F*_(3,232)_ = 2.335, *p* = 0.075	*F*_(3,232)_ = 2.890, *p = 0.036
E × S × P	*F*_(3,232)_ = 5.453, **p = 0.001	*F*_(3,232)_ = 1.037, *p* = 0.377	*F*_(3,232)_ = 1.540, *p* = 0.205

Statistical differences in mean length/dendrite, mean nodes/dendrite, and soma area were determined with three-way ANOVAs. Major effects: exposure (control chow vs prenatal ethanol exposure embryonic day 13.5–16.5), sex (male vs female), and postnatal day (P2, P4, P6, P8, P10, and P14), with interactions: E × S (exposure × sex), E × P (exposure × postnatal day), P × S (postnatal day × sex), and E × S × P (exposure × sex postnatal day). Statistics with *p* value ≤0.05 are bolded.

Prenatal ethanol exposure also differentially modified mean length/dendrite (µm; three-way ANOVA, exposure × sex × postnatal day: *p* = 0.001; Extended Data Fig. 7-1*A*, Table 7). SPNs from ethanol-exposed female mice displayed a decreased mean length/dendrite relative to SPNs from control-fed male and female mice at both P2 and P4–6. These differences in dendritic length/dendrite resolved completely by P14 (Extended Data Fig. 7-1*A*). An increase in the mean length of dendrites in SPNs from male mice exposed prenatally to ethanol relative to ethanol-exposed female mice was observed at P8–10, while decreased mean dendritic length persisted to P14 in SPNs in ethanol-exposed male mice (Extended Data Fig. 7-1*A*).

The effects of prenatal ethanol exposure on the mean of nodes per dendrite and soma area (µm^2^) were also sex-dependent (two-way ANOVAs, mean nodes per dendrite: exposure × sex: *p* = 0.008; soma area: exposure × sex: *p* = 0.002; Extended Data Fig. 7-1*C,D*, Table 7). We were unable to resolve differences in the mean number of dendritic branchpoints (nodes) per dendrite following prenatal ethanol exposure in male or female mice at specific postnatal time points (Extended Data Fig. 7-1*C*). Prenatal ethanol exposure resulted in increased the soma area (µm^2^) if SPNs from female mice, relative to those from control-fed F mice at P4, while ethanol exposure did not alter soma area in SPNs from male mice. No differences in soma area were observed at P2, P8–10, or P14 (Extended Data Fig. 7-1*D*, Table 7).

Overall, SPNs from both female and male mice demonstrate increases in dendritic complexity during the first two postnatal weeks with prenatal ethanol exposure. The increase in complexity is delayed in SPNs from male mice and result from changes to different aspects of dendritic growth with SPNs from male mice increasing dendritic length, while SPNs from female mice were observed to have an increased number of dendrites.

## Discussion

The clinical effects of prenatal ethanol exposure first become apparent during early childhood and persist into adolescence and adulthood. However, preclinical investigations of changes at the circuit level in animal models of prenatal ethanol exposure have frequently focused on a single developmental time point or population of neurons. Here, we explored the differential effects of prenatal ethanol exposure on two principal populations of striatal neurons: namely, GINs and SPNs, during the first two postnatal weeks, a period of dramatic changes in the functional properties of and synaptic inputs to striatal neurons as well as development of early motor behaviors. The major findings of the present study are as follows: (1) a brief binge-type prenatal exposure to ethanol affects the development of early motor behaviors, concurrent with the maturation of striatal GINs and SPNs in a sex-dependent manner, (2) striatal GINs and SPNs in male and female mice are differentially susceptible to prenatal ethanol exposure, and (3) the effects of prenatal ethanol exposure on both motor behaviors and neuronal maturation are dynamic and dependent upon timing during early development.

### Binge-type prenatal ethanol exposure results in sex-dependent differences in the development of early motor behaviors and the function of striatal neurons

Our results indicate that male neonates exposed prenatally to ethanol demonstrate developmental motor delays, evident in several motor tasks throughout the first two postnatal weeks independent of differences in gross physical development (Fig. 1*B*,*D–G*). Alternatively, changes in the motor development of female mice following prenatal ethanol exposure were less pronounced; subtle behavioral differences were reported in the onset of quadruped walking behavior and surface righting time (Fig. 1*D*,*F*). We also observed a period (P4–6) when female appeared to develop more rapidly than control-fed females in surface righting behavior (Fig. 1*F*).

Male vulnerability to deficits related to gestational and perinatal exposures, as well as neurodevelopmental disorders, is well documented (DiPietro and Voegtline, 2017; Bölte et al., 2023). While a number of cohort studies have identified higher rates of FASD in male children, they have not been recapitulated in recent epidemiological studies seeking to estimate the global prevalence of FASD (May et al., 1983, 2014; Astley, 2010; Thanh et al., 2014; Fox et al., 2015). However, the clinical presentation of FASD has been observed to differ between sexes, with individuals identifying as male demonstrating more dramatic differences in early development and measures of neurodevelopmental impairment, including the onset of early motor behaviors, while those identifying as female experienced higher rates of codiagnosed endocrine, mood, and anxiety disorders (May et al., 2017; Flannigan et al., 2023). Increased susceptibility of male offspring to the functional and behavioral effects of prenatal ethanol exposure has been observed in rodent models assessing adolescent and adult animals, including sex-dependent differences in both motor behaviors and the function of striatal neurons (Blanchard et al., 1993; Mooney and Varlinskaya, 2011; Rice et al., 2012; Rodriguez et al., 2016; Schambra et al., 2016; Rouzer and Diaz, 2022).

**Figure 8.**
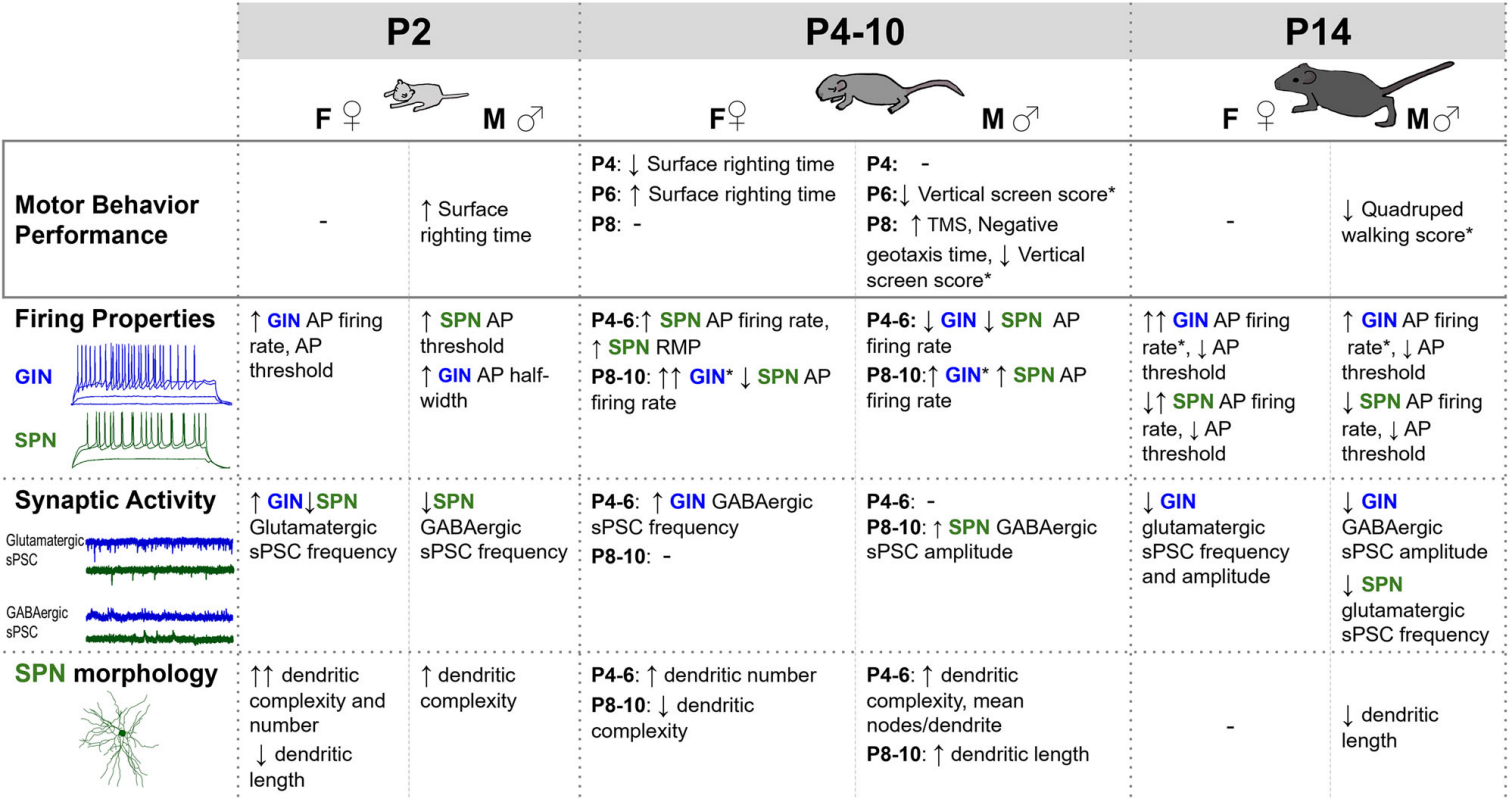
Prenatal ethanol exposure results in sex-dependent differences in early motor deficits coinciding with altered functional, synaptic, and morphological development of striatal neurons that vary with postnatal age. At P2, prenatal ethanol exposure results in a male-specific increase in surface righting time coinciding with significant sex differences in the effects of prenatal ethanol exposure on synaptic inputs to and functional properties of striatal GINs. In male mice exposed prenatally to ethanol, GIN demonstrate less increased AP half-width suggestive of a maturational delay, while SPNs receive fewer or weaker GABAergic synaptic inputs. In female mice, GINs show no differences in membrane properties but fire action potentials (APs) at a higher rate, while SPNs demonstrate no differences in GABAergic synaptic inputs and develop an increased dendritic complexity. However, SPNs from both male and female mice tended to have decreased glutamatergic synaptic innervation and had less mature/more excitable functional properties. At P4–6, prenatal ethanol exposure results in increased surface righting time only in female mice, while only male mice exposed prenatally to ethanol demonstrate increased negative geotaxis time and increased total motor score (TMS) at P8. Unlike female mice, male mice show fewer morphological differences early on (P2) but develop a longer lasting increase in dendritic complexity (P4–10), associated with longer, more highly branched dendrites. Differences in dendritic complexity coincided with changes in SPN AP firing rate that differ between female and male mice. Though both female and male mice demonstrate transient increases in SPN AP firing rate between P4 and P10, the increase observed at P4–6 in SPNs from female mice exposed prenatally to ethanol precedes a similar elevation in firing rate at P8–10 in SPNs from male mice with the same prenatal ethanol exposure (Fig. 4*A*). These changes in SPN firing properties occur simultaneously with increases in GABAergic activity: while at P4–6 the firing rate of striatal GINs from ethanol-exposed female mice does not differ from that of control-fed females, striatal GINs do display a higher GABAergic sPSC frequency (Figs. 4*A*, 6*B*). Alternatively, at P8–10, an increase in the amplitude of GABAergic sPSC frequency is increased in SPNs recorded from male mice exposed prenatally to ethanol (Fig. 6*C*). Data highlighted in blue represents a phenotype present in striatal GIN, while those highlighted in green represent a phenotype present in SPN. ↑ indicates a significant increase, ↓ indicates a significant decrease, - indicates no significant differences, * represents trend versus significant result.

The sex differences we observed in the effects of prenatal ethanol exposure on early motor behavior coincided with sex-dependent effects of prenatal ethanol exposure on the function and structure of striatal neurons at each developmental time point we investigated (Fig. 8). These data suggest a potential interplay between the sex-dependent morphological development of SPNs with prenatal ethanol exposure, excitability of and strength of GABAergic inputs to SPNs, consistent with previous data indicating that altered GABAergic input is related to both SPN firing and morphology in adult animals (Cuzon Carlson et al., 2020). However, the effects of prenatal ethanol exposure on motor development of female and male mice could also be due to altered function of other local and distant afferent inputs to the striatum, including those from cholinergic and dopaminergic neurons or altered development of other CNS structures that contribute to early motor behavioral development, which have been shown to demonstrate sexually dimorphic features (Miller, 1983; Blanchard et al., 1993; Boggan et al., 1996; Supekar and Menon, 2015; Giacometti and Barker, 2020; Zachry et al., 2021). The sex-dependent phenotypes we observe may also be related to sex differences in acute ethanol exposure on placental function, male gestational hormone levels, and postnatal gene expression related to epigenetic modification of sex chromosomes or arise from exacerbation of sex-dependent differences in development of striatal neurons or CNS immune cell function (McGivern et al., 1988; Bosco and Diaz, 2012; Schwarz et al., 2012; Lenz et al., 2013; Kleiber et al., 2014; Dorris et al., 2015; Cao et al., 2016; Chater-Diehl et al., 2017; Loke et al., 2018; Sutherland and Brunwasser, 2018; Kwan et al., 2020; Salem et al., 2021).

Alternatively, it remains possible that, given the subtlety of behavioral differences we observe, the sex differences in the development of early motor behaviors may result from natural variation observed both in the behavioral development of mice and the achievement of developmental milestones observed in infants and toddlers (Fox, 1965; Darrah et al., 1998; Valentini et al., 2019; Tupsila et al., 2022). This may be particularly relevant to behavioral analysis of female mice given our sample sizes were smaller for comparisons of female versus male mice (Table 2). The behavioral likely also reflect the effects of prenatal ethanol exposure on the development of other brain regions, particularly those within the cortico-basal ganglia-thalamic loop either resulting from or unrelated to the altered development of striatal neurons we demonstrated (Granato et al., 1995; Kozorovitskiy et al., 2012; El Shawa et al., 2013; Skorput et al., 2015; Miller, 2017; Louth et al., 2018; Delatour et al., 2019b). Alternatively, compensatory functional changes in other brain regions could explain times altered striatal neuronal function and morphology are present though the motor behaviors we assessed do not appear to be affected by prenatal ethanol exposure. Finally, we acknowledge that, as technical limitations required us to complete analysis of functional, synaptic development, morphological development at collapsed time points (P4–6, P8–10) rather than individual postnatal days, it is possible that significant differences in identified at these time points that were also masked or enhanced by the variation related to postnatal age, challenging comparisons with differences in motor development. Further investigation will be necessary to either rule out or incorporate alternative sources of sex differences in early motor behavior following prenatal ethanol exposure.

### Differential susceptibility of striatal GINs and SPNs to the effects of prenatal ethanol exposure during early postnatal development

We found that prenatal ethanol exposure differentially affected the functional and synaptic properties of striatal GINs and SPNs, adding to previous evidence that these two populations of neurons are differentially affected by exposures to ethanol in adult animals (Blomeley et al., 2011; Patton et al., 2016; Figs. 3–6). Overall, we observed events that facilitate the GABAergic signaling in the striatum, notably, increased excitatory inputs to striatal GINs, increased AP firing rate of striatal GINs, increased number or strength of GABAergic inputs to striatal GINs and SPNs, or differences in number of GABA receptors that facilitate larger amplitude GABAergic synaptic events, are more apparent in female mice exposed prenatally to ethanol, which also demonstrate fewer behavioral differences (Figs. 1*C–G*, 3*A–D*, 6*B*,*C*). Increases in GABAergic signaling are also apparent during periods of behavioral recovery in male mice, while evidence of diminished disrupted maturation of GABAergic signaling coincide with periods when prenatal ethanol exposure results in marked developmental motor delays in male mice (Figs. 1*C–G*, 3*A–D*, 6*B*,*C*).

Differences in the number, subtype, and function of GINs are frequently observed in developing circuits following early genetic or environmental insults and contribute to behavioral differences in neurodevelopmental disorders (Penzes et al., 2013; Ruden et al., 2021; Yang et al., 2021). It has previously been demonstrated that a brief binge exposure alters the migration of GINs to the developing cortex and contributes to an excitatory/inhibitory imbalance in the mPFC of young adult mice coinciding with hyperactivity and reversal learning deficits (Skorput et al., 2015). GABA facilitates early neuronal network development in a variety of ways: controlling gene expression, proliferation, growth, migration, synapse formation, and the coordinating firing of developing neurons (LoTurco et al., 1995; Wang and Kriegstein, 2008; Bortone and Polleux, 2009; Sernagor et al., 2010; Baho and Cristo, 2012). Further investigation is necessary to determine how prenatal ethanol exposure may contribute to differences in GABAergic signaling in the developing striatum.

Potential mechanisms contributing to observed differences in the effects of prenatal ethanol exposure on striatal GINs and SPNs include direct effects of ethanol on striatal neurons, indirect effects on the synaptic inputs to striatal neurons, or both. Recent investigation of the acute effects of ethanol on cortical cells in utero suggests that ethanol's effects on gene expression in embryonic neurons may be both subtype specific and sexually dimorphic (Salem et al., 2021; Sambo et al., 2022). Prenatal ethanol exposure could differentially modify the function or expression of ion channels in striatal GINs and SPNs, contributing to observed differences in membrane and firing properties (Figs. 3, 4; Extended Data Figs. 3-1, 4-1). Differences in the expression and subtype of potassium channels contributes to the maturation of firing properties in striatal GINs and SPNs and confer differential susceptibility to the effects of ethanol exposure in adult rodents (Lenz et al., 1994; Prüss et al., 2003; Plotkin et al., 2005; Brodie et al., 2007; Aryal et al., 2009; Dehorter et al., 2011; Tavian et al., 2011; Bates, 2013; Cazorla et al., 2014; Lieberman et al., 2018). Sodium and calcium channels can both contribute to the excitability of striatal neurons, are modified by ethanol exposure, and may differ between striatal neuronal subtypes during early development (Hunt and Mullin, 1985; Walter and Messing, 1999; Lee et al., 2022).

Prenatal ethanol exposure may also result in cell subtype-specific differences in afferents to striatal neurons contributing to differences in firing properties. Past work suggests that firing properties and synaptic inputs are highly related in striatal neurons; genetic manipulation of postsynaptic SPNs and their excitability can alter the strength of their glutamatergic inputs, while diminishing glutamatergic inputs to SPNs can modify their excitability (Kozorovitskiy et al., 2012; Mowery et al., 2017; Benthall et al., 2021). Striatal GINs and SPNs differ in their local and distant afferent connectivity and demonstrate different forms of short-term plasticity in response to excitatory stimulations depending source of afferents (Smith et al., 2004; Gittis et al., 2010; Lim et al., 2014; Clarke and Adermark, 2015; Arias-García et al., 2018; Johansson and Silberberg, 2020; Kocaturk et al., 2022). Prenatal ethanol exposure has been shown to alter the function of pyramidal neurons in the thalamus, as well as in motor, somatosensory, and prefrontal cortex (Granato et al., 1995; Mooney and Miller, 2010; Skorput et al., 2015; Delatour et al., 2019a,b; Mohammad et al., 2020). The function of postsynaptic glutamatergic AMPA and NMDA receptors is also differentially inhibited by ethanol exposure, and the relative density of these receptors also differs between striatal GINs and SPNs (Lovinger et al., 1989; Lovinger, 1993; Allgaier, 2002; Gittis et al., 2010; Möykkynen and Korpi, 2012; Vizcarra-Chacón et al., 2013). Striatal GIN and SPNs also vary in their GABAergic inputs, demonstrating differences in the strength and source of GABAergic inputs, their postsynaptic GABA receptor subunit expression, and the net effect of GABA to depolarize or hyperpolarize postsynaptic neurons (Misgeld et al., 1982; Dehorter et al., 2011; Straub et al., 2016; Boccalaro et al., 2019; Tapia et al., 2019).

### Dynamic change in the effects of prenatal ethanol exposure on early striatal development

Reports from longitudinal clinical imaging studies suggest that prenatal ethanol exposure can result in differential alterations the volume and functional connectivity of cortical structures and white matter, across development (Lebel et al., 2012; Treit et al., 2014; Hendrickson et al., 2018; Long et al., 2018; Kar et al., 2022; Moore and Xia, 2022). Our findings further indicate that developmental shifts in the effects of prenatal ethanol exposure during early development occur at the level of differences in the functional and morphological phenotypes of individual neurons (Figs. 3–7). We determined that while deficits in glutamatergic and GABAergic synapses were apparent at both P2 and P14, there was a transient resolution of early GABAergic and glutamatergic synaptic deficits from P4 to P10 that may occur via several possible mechanisms (Figs. 5, 6). Glutamatergic synaptic activity detectable in the embryonic striatum is likely driven by thalamic inputs which form E10–16.5, rather than those from the developing cortex which form later during embryonic and early postnatal development (Angevine, 1970; Molnár et al., 1998; Nakamura et al., 2005; Dehorter et al., 2011; Sohur et al., 2014). Thalamo- and corticostriate afferents also differ in the timing of their developmental shift in the contributions of postsynaptic AMPA and NMDA receptors to glutamatergic currents (Krajeski et al., 2019). Early postnatal glutamatergic currents result from spontaneous rather than AP-mediated vesicle release mechanisms and are largely NMDA receptor mediated, while AP and AMPA receptor-mediated events increase during the first three postnatal weeks (Mozhayeva et al., 2002; Dehorter et al., 2011; Krajeski et al., 2019). Considering these differences in the context of the timing of our in utero ethanol exposure, it is possible that the early changes we observed in glutamatergic synaptic inputs to SPN are due to alterations in the development of thalamic inputs, while the transient recovery we observed from P4 to P10 occurs due to formation of novel corticostriate connections, a developmental increase in thalamostriate activity or from compensatory increases in AP-mediated currents.

The nature of GABAergic signaling also changes in early development and may contribute to the temporal variation in the effects of prenatal ethanol exposure on developing striatal GINs and SPNs. In the cortex and hippocampus, early GABAergic action on postsynaptic GABA_A_ receptors results in a net depolarization of the postsynaptic neuron (Ben-Ari et al., 2012). This depolarizing action of GABA persists until the end of the first postnatal week, after which there is a developmental shift from depolarizing to hyperpolarizing action of GABA, which occurs due to differences in the concentration of intracellular chloride, mediated by changing expression levels of two chloride cotransporters: NKCC1 and KCC2 (Ben-Ari et al., 2012; Leonzino et al., 2016; Kalemaki et al., 2022). Alternatively, GABA_A_ receptor-mediated activity continues to result in depolarizing postsynaptic currents in SPN into adulthood due to a lack of KCC2 expression and may result in a net excitation or inhibition depending on SPN membrane potential (Misgeld et al., 1982; Bartos et al., 2007; Dehorter et al., 2009, 2011; Tapia et al., 2019). However, when during development the “GABA switch” occurs in striatal GINs and if SPNs demonstrate from excitatory to inhibitory action of depolarizing GABA are not yet known. Determining if and when these developmental changes in GABAergic signaling occur, and how they may be altered following prenatal ethanol exposure, will enhance our understanding of the differential effects of prenatal ethanol exposure on developing striatal neurons.

Additionally, though differences in performance on our series of motor tasks are nearly imperceptible between ethanol-exposed and control-fed mice by P14, we observed persistent differences in synaptic inputs to and firing properties of striatal neurons that differed between male and female mice (Figs. 1, 4*A*,*C*, 7, 8*C*). Specifically, while prenatal ethanol exposure resulted in more hyperpolarized AP thresholds and increased firing rates in striatal GINs from both female and male mice, GINs from female mice also demonstrated smaller amplitude GABAergic sPSCs, and SPNs from female mice demonstrated a decrease in the frequency and amplitude of glutamatergic sPSCs coinciding with current-dependent differences in firing rate (Figs. 4*A*,*C*,*E*,*G*, 7*E*,*F*, 8*C*). Alternatively, differences in firing rate following prenatal ethanol exposure were less pronounced in GINs from male mice and coincided with decreases in the frequency and amplitude of glutamatergic sPSCs as well as decreased AP firing rates and glutamatergic sPSC frequency in SPN (Figs. 4*E*,*G*, 7*E*). These data raise the question of how the effects of prenatal ethanol exposure on the function of striatal neurons may continue to evolve into adolescence and adulthood and ultimately contribute to differences in motor behavior in adolescents and adults diagnosed with FASD (Connor et al., 2006).

In conclusion, improving our understanding of the effects of prenatal ethanol exposure in the context of major events in neural development will be critical to interpreting the potential contribution of changes at circuit-level behavioral differences in individuals with FASD. Considering the changing impact of prenatal ethanol exposure across the developmental trajectory in the future may inform care management decisions for individuals with FASDs, beginning with early childhood diagnosis and intervention, extending into adulthood and across the lifespan.

## Synthesis

Reviewing Editor: Tod E Kippin, University of California Santa Barbara Decisions are customarily a result of the Reviewing Editor and the peer reviewers coming together and discussing their recommendations until a consensus is reached. When revisions are invited, a fact-based synthesis statement explaining their decision and outlining what is needed to prepare a revision will be listed below. The following reviewer(s) agreed to reveal their identity: NONE.

I have received two independent reviews of your manuscript both reviewers felt the study was interesting and generally well-conducted. The reviewers did have a few concerns which need to be addressed before a final decision can be reached on suitability of the manuscript for publication. I am, accordingly, recommending that you revise your manuscript.

Reviewer #1

The present manuscript tests the hypothesis that exposure to alcohol during gestation (~E15) in a mouse model will disrupt the intrinsic excitability and firing of striatal neurons leading to disruption of development of several motor behaviors expressed shortly after birth. The strength of the manuscript is that the authors examine the impact of alcohol exposure from neuronal activity and structure of two populations of striatal cells to the impact on motor behaviors that are thought to be reliant on these striatal circuits. The authors also investigate sex-dependent effects of alcohol in the model which is a major strength. Overall, the study is conducted well and the methods and results are clear. I only have a couple suggestions. First, given the large number of behaviors assessed, it would be nice to have a summary of the behavior effects somewhere in the text. This was done in later sections of the results when discussing firing characteristics. A similar summary could be provided for the behavior tests. Second, the discussion does a great job relating sex differences in motor behaviors to underlying changes in the function of striatal neurons. However, many studies have reported sex differences after developmental alcohol exposure, but few speculate as to why this might be the case. Is there a sex difference in how these striatal neurons and motor behaviors might be maturing that could underlie the observation here? It would be nice to see some speculation on this issue.

Reviewer #2

The manuscript describes outcomes of a 4 day exposure to alcohol on motor behavior in first 2 postnatal weeks, electrophysiology of striatum then analysis of dendritic morphology. The strength of the paper is the electrophysiology and morphology across this early developmental time window. The effects on behavior are not compelling and therefore neither are the comparisons of electrophysiology and behavior. How meaningful is it if there's a small effect on behavior on one day that is normal two days later and on all other days of testing? If the alcohol expousure is given in the 1st trimester, as stated by the authors, then changes seen early postnatally must be 2nd or 3rd trimester and with no behavioral effects at P14 the relevance is less. The discussion is long and might be improved by focusing on electrophysiology and morphology rather than on comparing these with behavor.

Is the electrophysiology done on the animals tested for behavior? If so, maybe direct comparisons could be made between outcomes for each animal. If not, then the comparison is brain outcomes in one animal with behavior in a different animal and this is not compelling.

If the electrophysiology underlies the behavior changes, how do the authors reconcile effects on electrophysiology and morphology at ages where there are no behavior changes?

There are "4-19 animals per group" but it is not clear what group sizes are for each outcome. Also clarify actual ages - there are 3 day windows for some behaviors and it's possible that what look like group differences are actually age differences, or at least that the age span influences outcome.

Results line 261 - "While no significant between group differences were identified at a single postnatal day in scoring of quadruped walking behavior, the most distinct between group differences were observed at P14... with fewer ethanol-exposed male mice demonstrating running behavior" - if this is based on number of males demonstrating running behavior those numbers or percents should be reported.

Line 301 "These data suggest that prenatal ethanol exposure can result in both improved performance (P4) and in deficits (P6) in surface righting behavior in female mice depending on the postnatal day." - because mice were chosen "randomly" for testing, the effects could also just be due to sampling variability.

Trends are reported in the Results section - it should be defined what is a trend.

Since Wald scores and ANOVA F values are reported in tables, they don't need to also be in the Results text -just reporting the p value in the text would make it easier to read.

In panel 3b DAPI is hard to see.

Include mouse background strain in abstract and methods.

## Author Response

Dr. Todd Kippin Reviewing Editor, eNeuro February 10, 2025 Dear Dr. Kippin We thank you and reviewers for the positive initial review of our manuscript titled: "Prenatal ethanol exposure results in cell-type, age, and sex-dependent differences in the neonatal striatum that coincide with early motor deficits," and the chance to submit a manuscript with revisions addressing the reviewer's comments (eN-NWR-0448-24).

We have carefully addressed each reviewer's comments point by point below, and made changes to the manuscript where indicated. We appreciate their thoughtful comments and believe the changes we have made have strengthened the findings we now present in our revised manuscript. Updates to the original manuscript are indicated by text highlighted blue.

Reviewer #1 Comment: Given the large number of behaviors assessed, it would be nice to have a summary of the behavior effects somewhere in the text. This was done in later sections of the results when discussing firing characteristics. A similar summary could be provided for the behavior tests.

Response: We thank the reviewer for this comment and have added a paragraph summarizing behavior effects in the Results section. (Results- Brief binge-type exposure to ethanol delays the development of motor behaviors in a sex-dependent manner). The summarized results of behavioral assays are also presented in a new summary figure described below (Figure 8).

Comment: The discussion does a great job relating sex differences in motor behaviors to underlying changes in the function of striatal neurons. However, many studies have reported sex differences after developmental alcohol exposure, but few speculate as to why this might be the case. Is there a sex difference in how these striatal neurons and motor behaviors might be maturing that could underlie the observation here? It would be nice to see some speculation on this issue.

Response: We thank the reviewer for this thoughtful observation, we now comment on multiple potential sources of the observed sex differences in the effects of prenatal ethanol exposure on the maturation of striatal medium spiny projection neurons to the discussion (Discussion- Binge-type prenatal ethanol exposure results in sex-dependent differences in the development of early motor behaviors and the function of striatal neurons).

Reviewer #2 Comment: How meaningful is it if there's a small effect on behavior on one day that is normal two days later and on all other days of testing? If the alcohol exposure is given in the 1st trimester, as stated by the authors, then changes seen early postnatally must be 2nd or 3rd trimester and with no behavioral effects at P14 the relevance is less. Line 301 "These data suggest that prenatal ethanol exposure can result in both improved performance (P4) and in deficits (P6) in surface righting behavior in female mice depending on the postnatal day." - because mice were chosen "randomly" for testing, the effects could also just be due to sampling variability.

Response: We thank the reviewer for these comments. Comparisons between human/rodent pre- and post-natal age are imperfect and based upon structural phenotypes rather than functional or behavioral development. The behavioral tasks presented here were chosen to attempt to approximate the gross and fine motor developmental milestones assessed in human infants and toddlers during the first six years of life. Given the rapid development of rodents relative to humans, we would hypothesize that developmental motor delays that play out over the course of weeks to months in human infants and toddlers could resolve over a series of days in mice. We posit that if presented with more complicated behavioral tasks at older postnatal ages, mice would likely continue to demonstrate differences in motor behavior at or after the last postnatal age we assessed (P14), as previously demonstrated by others using differing ethanol exposure paradigms. We would also argue that the subtle variability in behavioral phenotypes we observed is consistent with the variability in the presentation of fine and gross motor behavioral differences that has been observed in children affected by prenatal ethanol exposure. Future work should address the characteristics that are associated with specific vulnerability to the behavioral effects of prenatal ethanol exposure relative to littermates. Discussion of potential sources of variability in behavioral phenotype is now included (Discussion- Binge-type prenatal ethanol exposure results in sex-dependent differences in the development of early motor behaviors and the function of striatal neurons) and we have removed statements associating the timing of our prenatal ethanol exposure to a human gestational age.

Comment: The discussion is long and might be improved by focusing on electrophysiology and morphology rather than on comparing these with behavior.

Response: We have acknowledged potential limitations to our model system, softened the language and significantly limited discussion of the association between behavioral differences and altered electrophysiology and morphology in the Discussion section (Discussion- Binge-type prenatal ethanol exposure results in sex-dependent differences in the development of early motor behaviors and the function of striatal neurons). To balance Reviewer #2's concerns with Reviewer #1 positive review of the descriptions of the temporal association between behavioral, morphological and functional phenotypes, we now include a summary figure replacing the bulk of the text discussing this topic in the discussion section (Figure 8).

Comment: Is the electrophysiology done on the animals tested for behavior? If so, maybe direct comparisons could be made between outcomes for each animal. If not, then the comparison is brain outcomes in one animal with behavior in a different animal and this is not compelling.

Response: Electrophysiology was only completed on animals tested for behavior, additional animals were included in behavioral analysis but not all were included for electrophysiology given technical limitations. We agree that comparisons made between the results of behavioral testing for individual mice and the functional phenotypes would strengthen our supposition that the altered functional development of striatal neurons to differences in motor development we observed. However given the variability in both the functional and behavioral phenotypes we measured between individual animals, we do not have adequate sample sizes to achieve statistical power in order to complete this analysis. We have acknowledged that further work will be required to confirm the association between altered development of striatal GINs and differences in motor development in the discussion section (Discussion- Binge-type prenatal ethanol exposure results in sex-dependent differences in the development of early motor behaviors and the function of striatal neurons).

Comment: If the electrophysiology underlies the behavior changes, how do the authors reconcile effects on electrophysiology and morphology at ages where there are no behavior changes? Response: We thank the reviewer for this comment. At different developmental ages and with different exposure paradigms, prenatal ethanol exposure has been shown to result in the altered function of cortical projection neurons in the frontal, sensory and motor cortices as well as the thalamus. We now include discussion of the possibility that early altered development of striatal neurons may contribute to later dysfunction in cortico-basal ganglia- thalamic circuit that may contribute to differences in motor development that are preceded by but do not coincide with differences in the function of striatal neurons (Discussion- Binge-type prenatal ethanol exposure results in sex-dependent differences in the development of early motor behaviors and the function of striatal neurons).

Comment: There are "4-19 animals per group" but it is not clear what group sizes are for each outcome. Also clarify actual ages - there are 3 day windows for some behaviors and it's possible that what look like group differences are actually age differences, or at least that the age span influences outcome.

Response: We now include Table 1-2 demonstrating group sizes for each behavioral outcome. Analysis of behavioral differences by postnatal age are completed by postnatal day (control P2 vs. ethanol P2), while electrophysiological analysis was grouped (P2, P4-6, P8-10, P14). We now acknowledge in the discussion section that combining groups may mask or enhance differences related to normal functional, synaptic and morphological development with age. (Discussion- Binge-type prenatal ethanol exposure results in sex-dependent differences in the development of early motor behaviors and the function of striatal neurons).

Comment: Results line 261 - "While no significant between group differences were identified at a single postnatal day in scoring of quadruped walking behavior, the most distinct between group differences were observed at P14... with fewer ethanol-exposed male mice demonstrating running behavior" - if this is based on number of males demonstrating running behavior those numbers or percents should be reported.

Response: This was indeed based upon the number of males demonstrating running behavior. Percentages have been added (Results- A brief binge-type exposure to ethanol delays the development of motor behaviors in a sex-dependent manner).

Comment: Trends are reported in the Results section - it should be defined what is a trend.

Response: Trends were reported where p <0.070, we have now noted this in the methods section (Methods- Statistical Analysis). Where p>0.070 discussion of trends was removed from the results section (Results- Prenatal ethanol exposure differentially alters the maturation of active and passive properties of striatal neurons in male and female mice, Striatal GINs: Intrinsic electrical properties).

Comment: Since Wald scores and ANOVA F values are reported in tables, they don't need to also be in the Results text -just reporting the p value in the text would make it easier to read.

Response: We thank reviewer and have removed Wald scores and ANOVA F values represented in Tables 1-3, 2, 3, 4 and 5 from the text.

Comment: In panel 3b DAPI is hard to see.

Response: We thank the reviewer for this comment, the intensity has been adjusted to improve the contrast. We provide an updated figure (Figure 2b).

Comment: Include mouse background strain in abstract and methods.

Response: The mouse background strain is now included in the abstract and methods.
