# Supplementary figures and images for: Prenatal Ethanol Exposure Results in Cell Type, Age, and Sex-Dependent Differences in the Neonatal Striatum That Coincide with Early Motor Deficits
Source: eNeuro. 2025 Mar 25;12(3):ENEURO.0448-24.2025. doi: 10.1523/ENEURO.0448-24.2025 (PMC11949650; doi:10.1523/ENEURO.0448-24.2025)

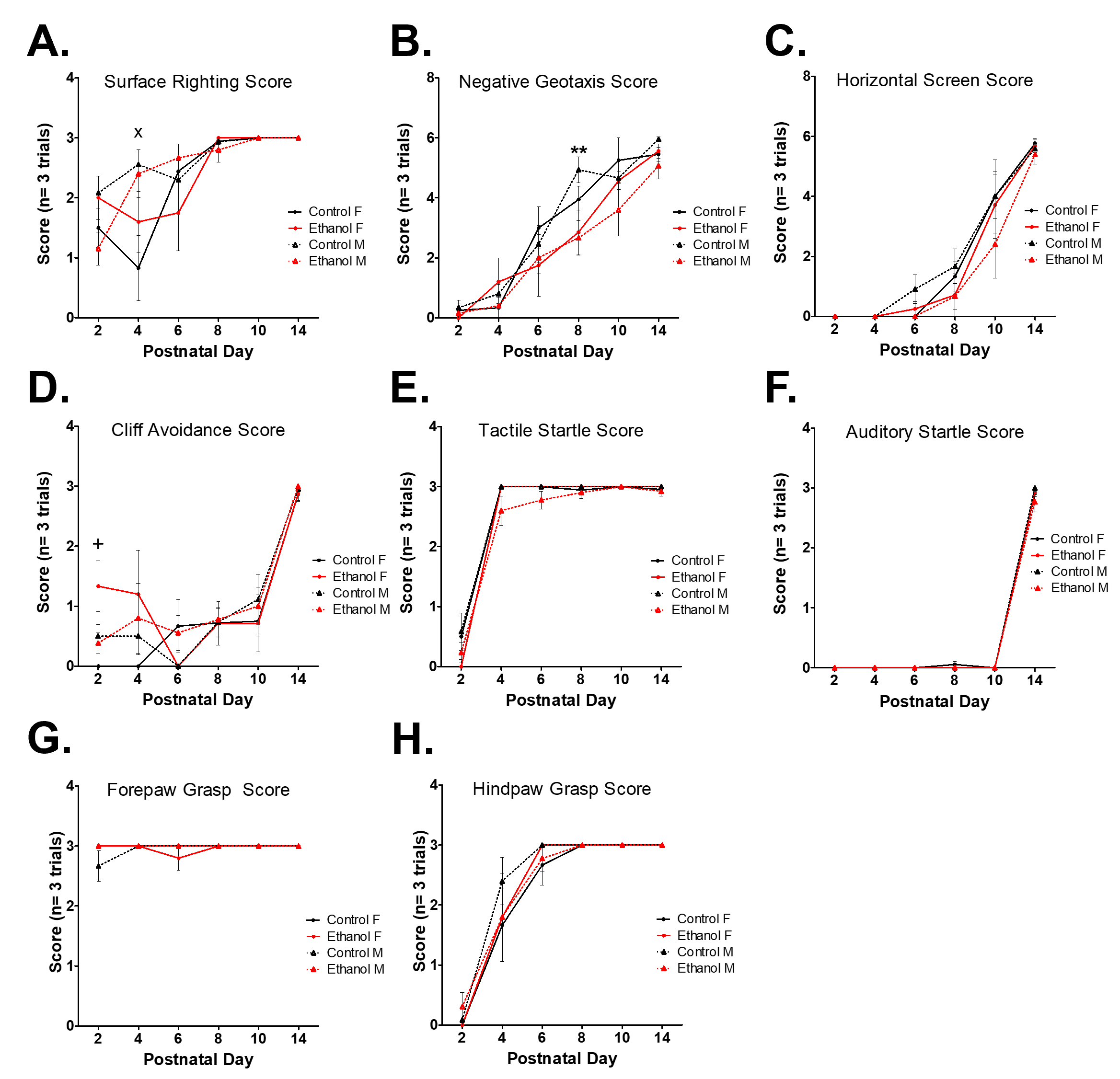

Supplement: Figure 1-1 — Prenatal ethanol exposure alters the development complex but not reflexive behaviors. Prenatal ethanol exposure results in significant differences in (A) surface righting score (B) negative geotaxis score (C) horizontal screen score, (D) cliff avoidance score, that depend on both sex and postnatal age (Kruskall-Wallis tests, surface righting score: P2: H(3) = 6.102, p = 0.107, P4: H(3) = 8.660, p = 0.034, Dunn’s post-hoc tests: ethanol F vs. control F: p=0.042, P6: H(3) = 3.176, p = 0.365, P8: H(3) = 0.796, p = 0.850, P10: H(3) = 0.000, p = 1.000, P14: H(3) = 0.000, p = 1.000; negative geotaxis score: P2: H(3) = 1.319, p = 0.725, P4: H(3) = 1.566, p = 0.667, P6: H(3) = 1.633, p = 0.652, P8: H(3) = 12.926, p = 0.005, Dunn’s post-hoc tests: ethanol M vs. ethanol F: P8: p= 0.007, P10: H(3) = 3.064, p = 0.382, P14: H(3) = 6.392, p = 0.094; horizonal screen score: P2: H(3) = 0.000, p = 1.000, P4: H(3) = 0.000, p = 1.000, P6: H(3) = 5.348, p = 0.148, P8: H(3) = 2.145, p = 0.543, P10: H(3) = 1.534, p = 0.674, P14: H(3) = 1.012, p = 0.798; cliff avoidance score: P2: H(3) = 9.160, p 0.027, Dunn’s post-hoc, control F vs. control M: P2: p=0.017, P4: H(3) = 3.190, p = 0.363, P6: H(3) = 5.861, p = 0.119, P8: H(3) = 0.159, p = 0.984, P10: H(3) = 1.250, p = 0.741, P14: H(3) = 5.279, p = 0.152). No differences were observed between groups in (E) tactile startle score, (F) auditory startle score, (G) forepaw grasp score, (H) hindpaw grasp score. Data are presented as mean score or time, error bars are standard error of the mean (SEM), **p<0.01, control male vs. ethanol male; +p<0.05, control male vs. control female; xp<0.05, ethanol male vs. ethanol female. Download Figure 1-1, TIF file. [file eneuro-12-ENEURO.0448-24.2025-s001.tif]

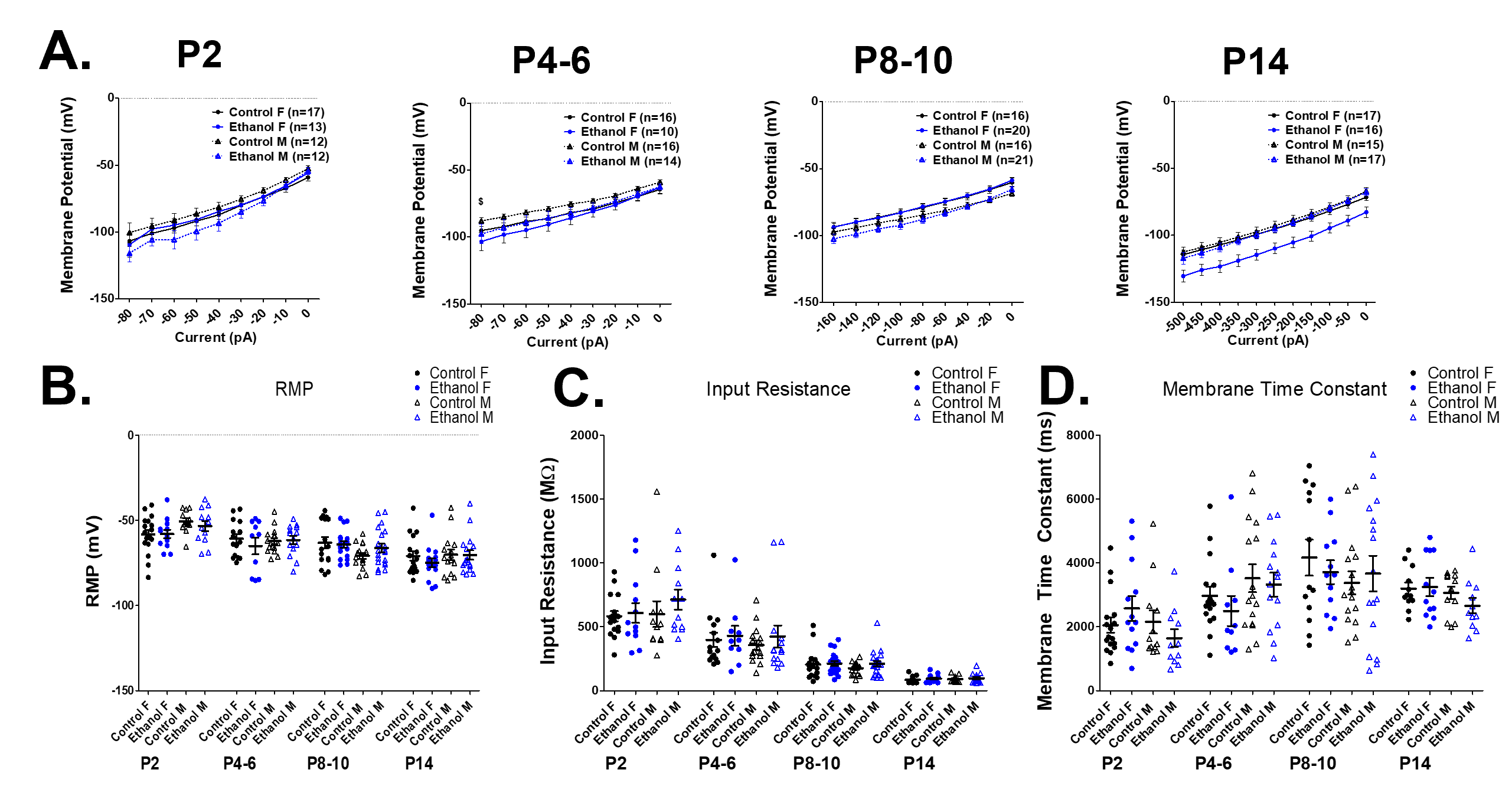

Supplement: Figure 3-1 — Prenatal ethanol exposure differentially effects the intrinsic properties of striatal GINs from female and male mice, depending on the postnatal day. (A) IV curves for responses to hyperpolarizing current steps during whole-cell current clamp recordings of striatal GINs during the first postnatal week (2-way ANOVAs, P2: group: F(3,450)= 7.015, p=0.=0001, current: F(8,450) = 63.35, p<0.001, group x current: F(24,450)=0.2834, p=0.9996; P4-6: group: F(3,468)= 11.35, p<0.001, current: F(8,468) = 38.60, p<0.001, group x current: F(24,468)=0.2114, p=1.000; P8-10: group: F(3,621)= 25.22, p<0.001, current: F(8,621) = 74.29, p<0.001, group x current: F(24,621)=0.2189, p=0.9890; P14: group: F(3,671)= 15.53, p<0.001, current: F(10,671)= 83.66, p<0.001, group x current: F(30,671)=0.08655, p=1.000. (B) Prenatal ethanol exposure resulted in sex-dependent differences in resting membrane potential (RMP) that varied based on the postnatal day, though no significant differences were observed between groups on individual postnatal days (1 way ANOVAs, P2: F(3,50)= 2.008, p=0.125, P4-6: F(3,50)= 1.293, p=0.287, P8-10: F(3,69)= 2.244, p=0.091, P14: F(3,61)=0.642, p=0.591). (C) Input resistance (IR) (mΩ) and (D) membrane time constant (ms) were unaffected in striatal GINs were unaffected by prenatal ethanol exposure, sex or postnatal day (1-way ANOVAs, IR: P2: F(3,50) = 0.611, p =0.649 P4-6: F(3,50) = 0.219, p =0.883; P8-10: F(3,69) = 0.557, p =0.302; P14: F(3,61) = 0.611, p =0.610; membrane time constant: P2: F(3,50) = 1.318, p=0.279; P4-6: F(3,61) = 0.689, p =0.563; P8-10: F(3,69) = 1.239, p =0.302; P14: F(3,61) = 1.277, p =0.294). Data are presented as means (bars), error bars are standard error of the mean (SEM), dots are individual neurons from at least 3 animals per group. $p<0.05, control male vs. ethanol female. Download Figure 3-1, TIF file. [file eneuro-12-ENEURO.0448-24.2025-s002.tif]

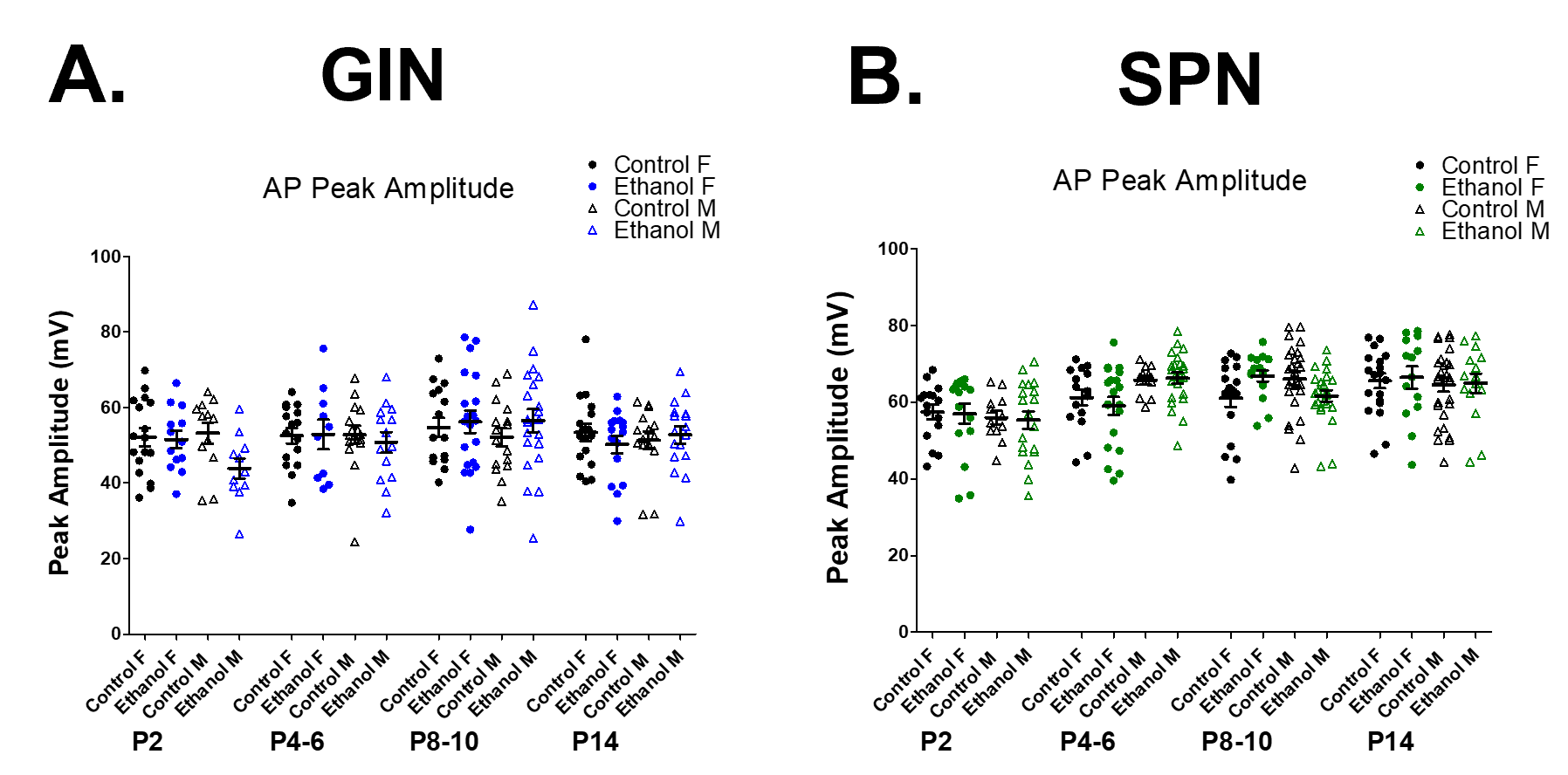

Supplement: Figure 3-2 — Prenatal ethanol exposure does not alter the action potential (AP) amplitude of developing striatal GABAergic interneurons (GINs) or striatal projection neurons (SPNs). (A) The peak amplitude of APs (pA) did not differ in striatal GINs or (B) SPNs following prenatal ethanol exposure. Data are presented as means (bars), error bars are standard error of the mean (SEM), dots are individual neurons from at least 3 animals per group. Download Figure 3-2, TIF file. [file eneuro-12-ENEURO.0448-24.2025-s003.tif]

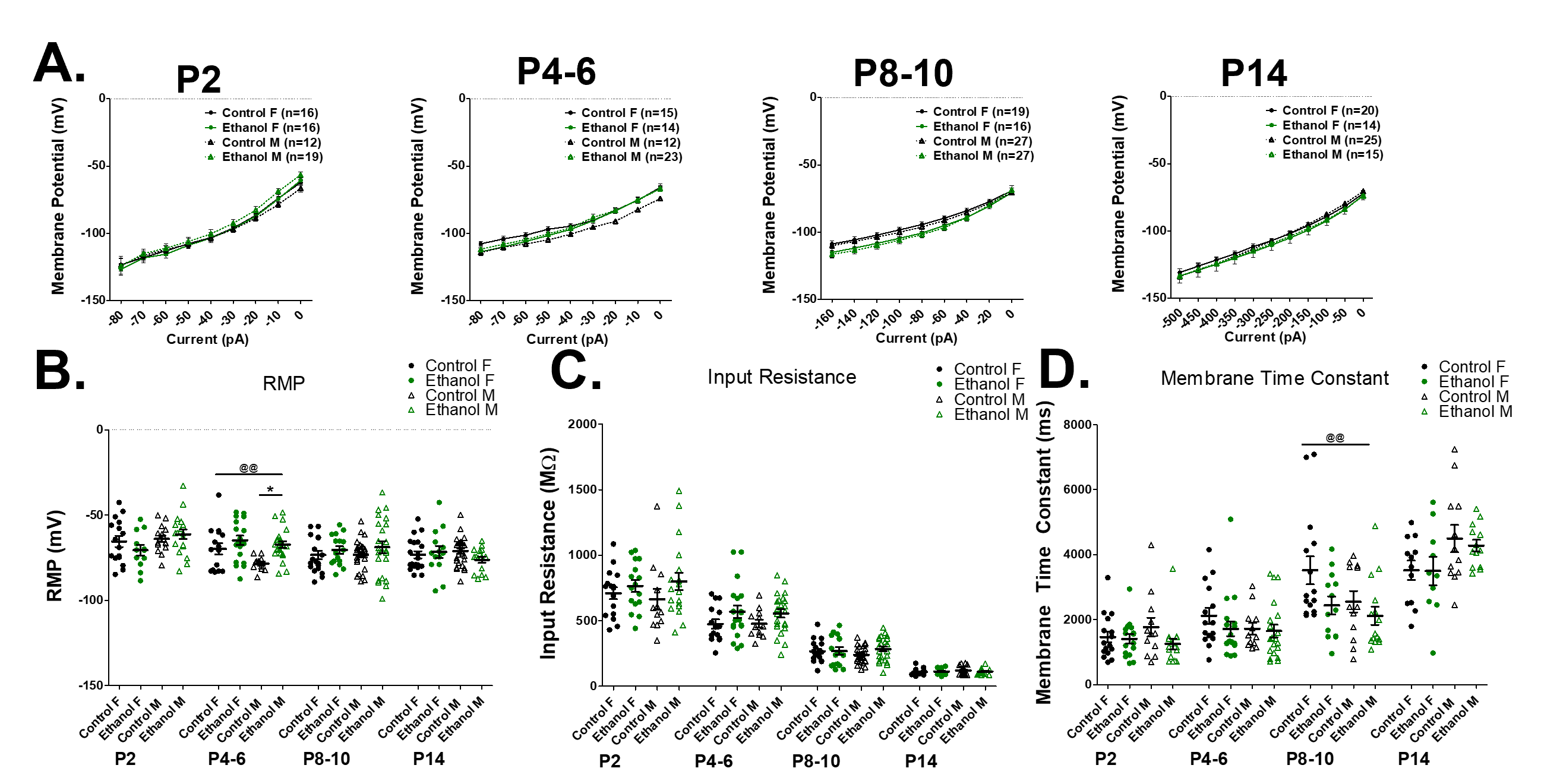

Supplement: Figure 4-1 — Prenatal ethanol exposure differentially effects the intrinsic properties of striatal SPNs from female and male mice, depending on the postnatal day. (A) The effects of prenatal ethanol exposure on IV curves for responses to hyperpolarizing current steps during whole-cell current clamp recordings of SPNs during the first postnatal week vary by group (2-way ANOVAs, P2: group: F(3,531)= 3.047, p=0.0284, current: F(8,531) = 151.8, p<0.001, group x current: F(24, 531)=0.2490, p=0.9999; P4-6: group: F(3,540)= 13.10, p<0.001, current: F(8,540) = 169.0, p<0.001, group x current: F(24, 540)=0.4369, p=0.9999; P8-10: group: F(3,765)= 14.67, p<0.001, current: F(8,765) = 164.9, p<0.001, group x current: F(24,765)=0.4551, p=0.9890; P14: group: F(3,770)= 3.537, p=0.0145, current: F(10,770) = 243, p<0.001, group x current: F(30,770)=0.1753, p=1.000). (B) Prenatal ethanol exposure resulted in sex-dependent differences in SPN RMP that varied based on the postnatal day: At P4-6: prenatal ethanol exposure resulted in significantly depolarized RMP in male mice relative to control-fed male and female mice (1-way ANOVA: F(3,66) = 4.632, p=0.005, Bonferroni post-hoc tests: ethanol M vs. control F: p=0.023, ethanol M vs control F: p=0.004). SPN RMP was unaltered by prenatal ethanol exposure at P2, 8-10 or P14 (1-way ANOVAs, P2: F(3,59) = 1.790, p =0.159; P8-10: F(3,81) = 0.796, p =0.499; P14: F(3,73) = 1.042, p = 0.379). (C) Prenatal ethanol exposure did not alter the IR of SPNs (1-way ANOVAs, P2: F(3,59) = 0.946, p =0.424; P4-6: F(3,66) = 1.039, p =0.202; P8-10: F(3,81) = 1.182, p =0.322; P14: F(3,73) = 1.034, p=0.383). (D) Prenatal ethanol exposure results in decreased membrane time constant in SPNs from ethanol-exposed M mice at P8-10, relative to control-fed F mice, but did not alter membrane time constant in F mice. (1-way ANOVAs, P2: F(3,59) = 1.175, p =0.327; P4-6: F(3,66) =0.934, p =0.430; P8-10: F(3,81) = 3.405, p =0.024; P14: F(3,73) = 2.283, p = 0.093. Data are presented as mean [file eneuro-12-ENEURO.0448-24.2025-s004.tif]

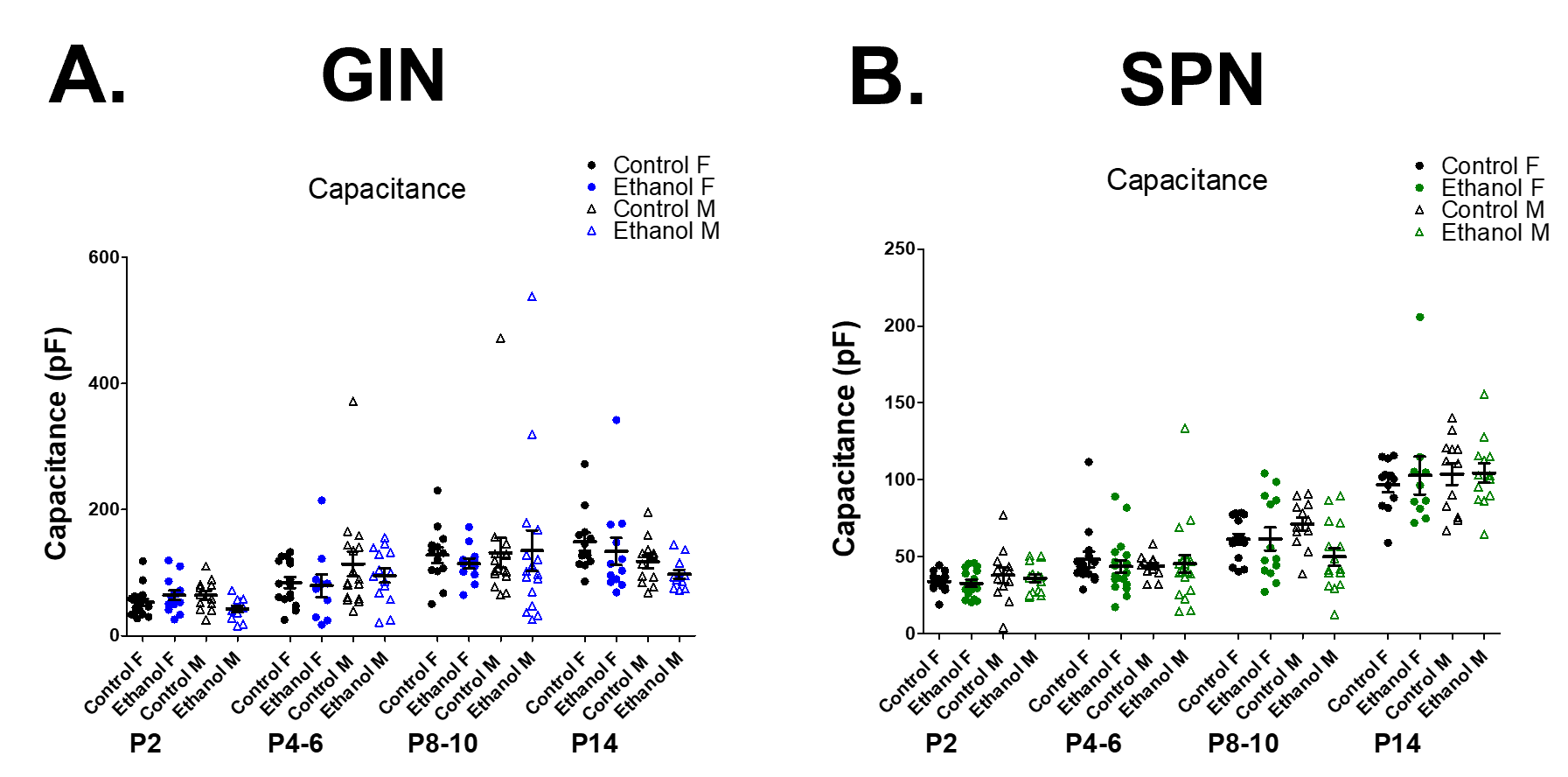

Supplement: Figure 4-2 — Prenatal ethanol exposure does not alter membrane capacitance of developing striatal GABAergic interneurons (GINs) or striatal projection neurons (SPNs) (A) Membrane capacitance was unaffected by prenatal ethanol exposure in striatal GINs or (B) SPNs. Data are presented as means (bars), error bars are standard error of the mean (SEM), dots are individual neurons from at least 3 animals per group. Download Figure 4-2, TIF file. [file eneuro-12-ENEURO.0448-24.2025-s005.tif]

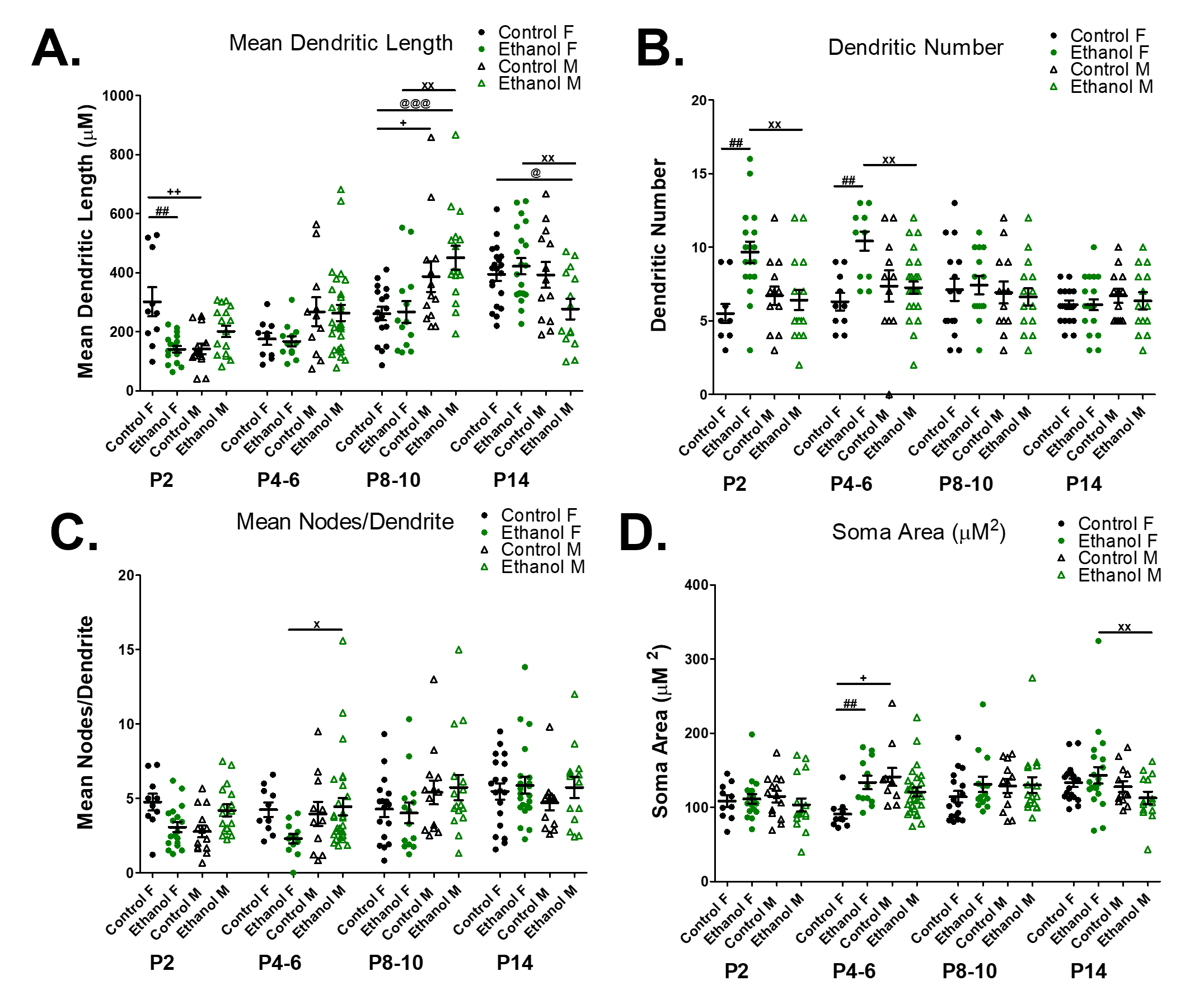

Supplement: Figure 7-1 — Prenatal ethanol exposure result in early postnatal increases in spiny projection neuron (SPN) dendritic morphology: length, number, branching, and soma area. (A) Prenatal ethanol exposure decreased the mean length/dendrite (µM) in SPN F mice relative to control-fed F while control-male mice also displayed significantly decreased mean length/dendrite relative to control fed F at P2, while prenatal ethanol exposure resulted in no significant differences at P4-6. At P8-10 prenatal ethanol exposure increased the mean length/dendrite in SPNs from M mice relative to ethanol-exposed and control-fed F mice, control-fed male mice also displayed significantly increased mean length/dendrite relative to control-fed F mice. At P14, SPNs from ethanol-exposed M mice relative displayed a decreased mean length/dendrite relative to ethanol-exposed and control-fed F mice. (P2: (Kruskall-Wallis test, H(3) = 13.49, p=0.0039, Dunn’s post-hoc tests: ethanol F vs. control F: p<0.05, ethanol F vs. control M, p>0.05; P4-6: Kruskal-Wallis test, H(3) = 6.905, p=0.0750; P8-10: one-way ANOVA, F(3,59) = 6.276, p = 0.001, Bonferroni post-hoc tests: ethanol M vs. ethanol F, t= 3.427, p<0.01, ethanol M vs. control F, t=3.710, p<0.01; P14: one-way ANOVA, F(3,64) = 3.962, p = 0.0118, Bonferroni post-hoc tests: ethanol M vs. ethanol F, t= 3.308, p<0.01). (B) Prenatal ethanol exposure results in a transient increase in the number of dendrites in SPNs from P2 and P4-6 F mice relative to those from control-fed F and ethanol-exposed M mice of the same ages, that resolves by P8-10 (Kruskal-Wallis tests, P2: H(3)= 12.832, p=0.005, Dunn’s post-hoc tests: ethanol F vs. control F: p< 0.05, ethanol F vs. ethanol M, p<0.05; P4-6: H(3) = 14.116, p=0.003, Dunn’s post-hoc tests: ethanol F vs. control F: p<0.01, ethanol F vs. ethanol M, p<0.01; P8-10: H(3)= 0.897, p=0.826; P14: H(3)= 0.747, p=0.862). (C) Prenatal ethanol exposure resulted in trend towards a decreased mean number of nodes/dendrite in SPNs from P4-6 F [file eneuro-12-ENEURO.0448-24.2025-s006.tif]
